# SAXS Reveals the Stabilization Effects of Modified Sugars on Model Proteins

**DOI:** 10.3390/life12010123

**Published:** 2022-01-15

**Authors:** Astra Piccinini, Eva C. Lourenço, Osvaldo S. Ascenso, Maria Rita Ventura, Heinz Amenitsch, Paolo Moretti, Paolo Mariani, Maria Grazia Ortore, Francesco Spinozzi

**Affiliations:** 1 Department of Life and Environmental Sciences, Polytechnic University of Marche, 60131 Ancona, Italy; a.piccinini@pm.univpm.it (A.P.); p.moretti@pm.univpm.it (P.M.); p.mariani@univpm.it (P.M.); m.g.ortore@univpm.it (M.G.O.); 2 Extremochem, Rua Ivone Silva, 1050-124 Lisboa, Portugal; eva.lourenco@extremochem.com (E.C.L.); osvaldo.ascenso@extremochem.com (O.S.A.); 3 Instituto de Tecnologia Química e Biológica António Xavier, Universidade Nova de Lisboa, 2780-157 Oeiras, Portugal; rita.ventura@extremochem.com; 4 Institute for Inorganic Chemistry, Graz University of Technology, 8010 Graz, Austria; amenitsch@tugraz.at

**Keywords:** small-angle X-ray scattering, protein stabilization, solvation, thermodynamic model, myoglobin, insulin

## Abstract

Many proteins are usually not stable under different stresses, such as temperature and pH variations, mechanical stresses, high concentrations, and high saline contents, and their transport is always difficult, because they need to be maintained in a cold regime, which is costly and very challenging to achieve in remote areas of the world. For this reason, it is extremely important to find stabilizing agents that are able to preserve and protect proteins against denaturation. In the present work, we investigate, by extensively using synchrotron small-angle X-ray scattering experiments, the stabilization effect of five different sugar-derived compounds developed at ExtremoChem on two model proteins: myoglobin and insulin. The data analysis, based on a novel method that combines structural and thermodynamic features, has provided details about the physical-chemical processes that regulate the stability of these proteins in the presence of stabilizing compounds. The results clearly show that some modified sugars exert a greater stabilizing effect than others, being able to maintain the active forms of proteins at temperatures higher than those in which proteins, in the absence of stabilizers, reach denatured states.

## 1. Introduction

Over recent decades, protein therapeutics have increased significantly, owing to their positive effects in the treatments of several diseases. The first human protein therapeutic that was introduced was human insulin, derived from recombinant DNA techniques. Proteins, among other small-molecules drugs, can perform complex functions that reduce the drug toxicity and the immune response, because they are naturally produced by the human body. Additionally, they have the most dynamic role of all the body macromolecules and the biggest influence in terms of clinical utility [1]. Proteins are extensively used in the treatment of several diseases, including cancer, HIV and diabetes. In this context, monoclonal antibodies, cytokines and interferons are just a few examples of the wide range of proteins that can be used as therapeutics macromolecules [2]. However, there are a lot of limitations concerning the protein therapeutic strategy. First of all, they are very expensive due to their expensive production cost, and this may limit their use in the global market. Secondly, they need to be stored and transported by maintaining a cold regime, in order to preserve their native structures, since a conformational change may result in a loss of their activity. The degradation mechanisms that usually occur can involve both physical or chemical processes. Denaturation, noncovalent and covalent aggregation, deamination and oxidation caused by heat, chemical factors or other types of stresses can indeed provoke the loss of the three-dimensional structure of a protein. The hydrophobic patches of a protein are usually folded inward when the macromolecule is in its native state, whereas they can be exposed to the solvent during unfolding processes. As a consequence, the increase in the available surface area intensifies the risk of adsorption and aggregation [3]. For all these reasons, it is of great importance to find good ways to preserve proteins at a temperature as close as possible to room temperature, and one of the best solutions is to use low molecular weight, chemically unreactive stabilizer compounds. These stabilizers can encompass a wide variety of molecules including sugars, salts, amino acids, and polymers such as polyols and polyethylene glycols [4]. Stabilizers are used in many technological fields, from biology to engineering [5]. The food industry, for example, is an important field where stabilizers have reached a high resonance. Additives are largely employed to maintain the physical stability of products, discouraging deteriorating processing that can damage food [6]. Additionally, in biology, stabilizers are one of the most important sources that can be used to preserve proteins against denaturation, which often occurs because of several denaturing factors such as chemicals, high temperature, high pressure, and non-physiological pH. These extreme factors are able to modify the native protein conformation, which is stabilized by a network of intramolecular hydrogen bonds, salt bridges and van der Waals interactions, as well as by the interactions with water and other molecules in solution [7]. One of the main groups of compounds that are used for stabilizing proteins are sugars, which are able to increase the energy barriers between folded and unfolded states of a protein [8]. It has been shown that sugars do not interact directly with the protein surface, but they can trap the water molecules in solution around the protein to preserve its hydration shell and maintain its stability [9,10]. The major driving forces that are involved in protein stabilization are considered to be the hydrogen bonds, which take place between the protein and the water molecules that surround the protein shell [8].

In this work, we focused on five synthesized sugars (hereafter referred to as modified sugar) developed by ExtremoChem. ExtremoChem has developed several new stabilizers [11] based on known osmolytes [12] that are able to stabilize biomolecules, including nucleic acids and proteins, against stresses such as temperature excursions, shaking and other mechanical stresses, high and low pH values and high concentration. In our experiment, we tested these stabilizers on myoglobin and insulin at increasing temperatures, with the aim to examine their stabilization properties. Samples were investigated by a synchrotron small-angle X-ray scattering (SAXS) technique and data were analyzed in terms of the distribution of proteins in different states (monomers, dimers, tetramers, and hexamers, just for insulin), considering long-range protein–protein interactions and by employing multimeric equilibrium processes in combination with exchange equilibrium processes between modified sugar and water molecules that occur over the surface of individual protein states. As a result, we were able to quantify the stabilizing effect of the five modified sugars regarding each state of the model proteins myoglobin and insulin.

## 2. Materials and Methods

### 2.1. Sample Preparation

Myoglobin (MB) from equine heart was purchased from Sigma-Aldrich and used at concentrations of 2 and 10 g/L dissolved in 10 mM phosphate buffer at pH 5. Insulin (IN) from a bovine pancreas was prepared at 2 g/L and dissolved in the same buffer at pH 3. Five synthesized modified sugars, (EC101, EC202, EC212, EC311 and EC312), made by the Portuguese chemical synthesis company ExtremoChem, were dissolved in the protein solutions at three different final concentrations: 0.05, 0.1, and 0.25 M. These modified sugars contain a mannose, glucose or galactose moiety with different substituents at the anomeric position [11], which can be charged or neutral. Two modified sugars, EC101 and EC202, form ionic species when dissolved in water. Moreover, they were found to slightly increase the pH values of the solutions (see Table S1 in the Supplementary Materials).

### 2.2. SAXS Experiments

SAXS experiments were performed at the Austrian SAXS beamline of Elettra synchrotron (Trieste, Italy) [13]. Measurements were carried out at $25^{\circ }$, $35^{\circ }$ and $60^{\;\circ }$C for myoglobin, whereas the same analysis was performed at $25^{\circ }$, $30^{\circ }$, $35^{\circ }$, $40^{\circ }$, $45^{\circ }$, $55^{\circ }$ and $60^{\;\circ }$C for insulin. Over the course of the experiment, the new μ-Drop sample changer recently developed in the Austrian beamline was used [14]. The modulus *q* of the scattering vector, related to the scattering angle 2θ and to the X-ray wavelength λ=1.54 Å by the relationship q=(4π/λ)sinθ, was fixed between 0.01 and 0.35 Å${}^{-1}$. For each sample, twelve bidimensional and isotropic SAXS patterns were collected by a Pilatus3 1M detector and subsequently treated with FIT2D [15] to apply the beamstop and detector mask and to perform the radial average. Finally, by using the SAXS data reduction system (SAXS dog) for the subtraction of the buffers isotropic SAXS signal from the one of the samples, the normalization to the intensity of the primary beam and the correction for the samples’ transmissions, the experimental macroscopic scattering cross section $\frac{d\Sigma }{d\Omega }\left(q\right)$ of each sample was obtained.

### 2.3. SAXS Data Analysis

The analysis of SAXS data was performed by assuming that proteins in solution can be present in $N_{s}$ different *states* (e.g., folded oligomers or unfolded chains), without any preferential orientation, and that long-range isotropic protein–protein interactions may occur. In these circumstances, the macroscopic differential scattering cross section (the precious information provided by SAXS experiments) can be written as

(1) $$\begin{array}{c} \frac{d\Sigma }{d\Omega }\left(q\right)=n\;P\left(q\right)\;S_{M}\left(q\right). \end{array}$$

This equation contains three relevant factors. Firstly, *n* is the nominal number density of the protein monomers, simply related to the w/v protein concentration, *c*, through Avogadro’s number, $N_{A}$, and the monomer molecular weight, $M_{1}$, by $n=cN_{A}/M_{1}$. The second term, P(q), is the so-called *effective form factor*,

(2) $$\begin{array}{ccc} P(q) & = & \sum_{j=1}^{N_{s}}\frac{x_{j}}{\alpha_{j}}P_{j}\left(q\right) \end{array}$$

where $P_{j}\left(q\right)$ is the form factor (the orientational average of the squared excess X-ray scattering amplitude) of the *j*-protein state, $\alpha_{j}$ is the corresponding aggregation number, whereas $x_{j}$ is the molar fraction of nominal protein monomers that are forming the *j*-state, with the condition

(3) $$\begin{array}{c} \sum_{j=1}^{N_{s}}x_{j}=1 \end{array}$$

The third term, $S_{M}\left(q\right)$, is known as the *measured structure factor* and depends on the average protein–protein structure factor, S(q), according to $S_{M}\left(q\right)=1+\beta \left(q\right)\left[S\left(q\right)-1\right]$, where β(q) is the coupling function, with $\beta \left(q\right)=|P^{\left(1\right)}{\left(q\right)|}^{2}/P\left(q\right)$ and $P^{\left(1\right)}\left(q\right)$ being the weighted average of the orientational average of the excess X-ray scattering amplitude $P_{j}^{\left(1\right)}\left(q\right)$ of the *j*-state

(4) $$\begin{array}{ccc} P^{\left(1\right)}\left(q\right) & = & \sum_{j=1}^{N_{s}}\frac{x_{j}}{\alpha_{j}}P_{j}^{\left(1\right)}\left(q\right). \end{array}$$

The calculation of both $P_{j}\left(q\right)$ and $P_{j}^{\left(1\right)}\left(q\right)$ was carried out on the basis of protein data bank (PDB, [16]) atomic structure associated with the *j*-state by using the SASMOL approach [17]. This method is based on the description of the solvent molecules in contact with the protein as dummy Gaussian spheres and determines the number and the geometrical coordinates of such spheres by burying the protein in a tetrahedral close-packed (TCP) lattice of dummy spheres. Consequently, the number and the positions of the water molecules can be obtained in the first $N_{sh}$ hydration shells of the *j*-protein state and a scattering length density (SLD) that can differ from the one of the bulk solvent is assigned to each of them. Typically, the thickness of each water shell is considered to be equal to 2.8 Å. Notice that in this work we have considered $N_{sh}=2$. This feature is particularly useful in the presence of a binary solvent, such as a solution of water and modified sugar, where preferential solvation effects can lead to a modification of the composition of the binary solvent in contact with the protein surface with respect to the composition of the bulk binary solvent.

#### 2.3.1. Multimeric Equilibrium Processes in Binary Solvents

**Figure 1:**
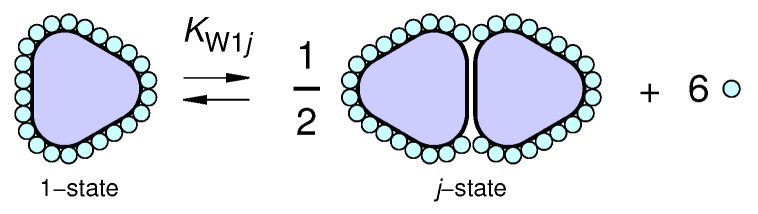
Sketch of an equilibrium process of the protein in water from the monomeric (1-)state to the dimeric (*j* = 2)-state (scheme (6), with $\alpha_{j}=2$ and $m_{1}-\alpha_{j}^{-1}m_{j}=6$).

**Figure 2:**
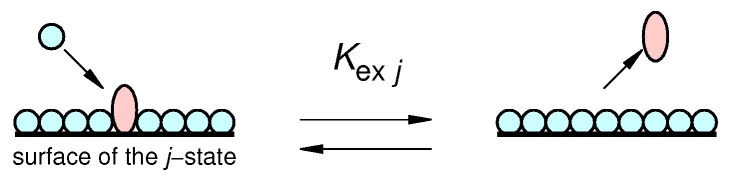
Sketch of the water-cosolvent (blue spheres and red ellipsoids, respectively) exchange equilibrium process over the surface of the *j*-protein state.

In equilibrium conditions, the distribution of proteins in the $N_{s}$ states and the composition of the first protein hydration shell as a function of protein concentration, solvent composition and temperature can be determined by considering the interplay of different elementary processes. *First*, we consider the process of transformation of a protein (hereafter indicated by the symbol P) dissolved in water at a certain pH and at a certain ionic strength (*I*) from the state 1 (which is assumed to be a monomeric state, typically a native state) to the state *j*,

(5) $$\begin{array}{ccc} & & P_{1}{\left({W_{s}}_{1}\right)}_{m_{1}}⇌\alpha_{j}^{-1}P_{j}{\left({W_{s}}_{j}\right)}_{m_{j}}+\left(m_{1}-\alpha_{j}^{-1}m_{j}\right)W_{b} \end{array}$$

where $m_{j}$ is the number of water sites in the first hydration shell of the *j*-state (a value that can be determined by SASMOL), ${W_{s}}_{j}$ represents a water molecule attached to the surface of the protein in the *j*-state and $W_{b}$ represents a water molecule in the bulk (see Figure 1). By assuming an ideal thermodynamic behavior of the system, the equilibrium constant $K_{W1j}$ as well as the standard Gibbs free energy change $\Delta G_{W1j}$ associated with this process is

(6) $$\begin{array}{ccc} & & K_{W1j}=\frac{C_{P_{j}{\left({W_{s}}_{j}\right)}_{m_{j}}}^{\alpha_{j}^{-1}}X_{W_{b}}^{m_{1}-\alpha_{j}^{-1}m_{j}}}{C_{P_{1}{\left({W_{s}}_{1}\right)}_{m_{1}}}}=e^{-\Delta G_{W1j}/\left(RT\right)} \end{array}$$

where the symbol *C* is the molar concentration (used for the solutes, with C=1 M being their standard state) and the symbol *X* stands for the molar fraction (used of the solvent (water), with X=1 being its standard state). The *second* process refers to proteins dissolved in a binary solvent constituted by water and a cosolvent (such as a modified sugar) and describes the exchange of a cosolvent molecule attached to the first hydration shell of the protein in the *j*-state (indicated by the symbol ${G_{s}}_{j}$) with a bulk water molecules (see Figure 2 for a clarifying example),

(7) $$\begin{array}{ccc} & & {G_{s}}_{j}+W_{b}⇌G_{b}+{W_{s}}_{j} \end{array}$$

that leads to the formation of a cosolvent molecule in the bulk ($G_{b}$) and a water molecule in the first shell (${W_{s}}_{j}$). According to the well-established Schellmann model [18,19,20,21], this *exchange equilibrium* has been found to be simply described by the thermodynamic constant $K_{\mathrm{ex}j}$ and the related standard Gibbs free energy change $\Delta G_{\mathrm{ex}j}$,

(8) $$\begin{array}{ccc} & & K_{\mathrm{ex}j}=\frac{\phi_{j}X_{G_{b}}}{\left(1-\phi_{j}\right)X_{W_{b}}}=\frac{\phi_{j}x_{G_{b}}}{\left(1-\phi_{j}\right)\left(1-x_{G_{b}}\right)}=e^{-\Delta G_{\mathrm{ex}j}/\left(RT\right)} \end{array}$$

where $\phi_{j}$ is the fraction of first hydration shell sites in the protein *j*-state occupied by water molecules. We have introduced the molar fraction of cosolvent in the bulk binary solvent,

(9) $$\begin{array}{ccc} & & x_{G_{b}}=\frac{X_{G_{b}}}{X_{G_{b}}+X_{W_{b}}} \end{array}$$

To note, if water is preferentially attached to the protein, $K_{\mathrm{ex}j}>1$ ($\Delta G_{\mathrm{ex}j}<0$), otherwise, when there is a preferential binding of protein with cosolvent molecules, $K_{\mathrm{ex}j}<1$ ($\Delta G_{\mathrm{ex}j}>0$). We assume that the exchange equilibrium processes are independent events, so that the probability that *n* water sites are occupied by water molecules and the remaining $m_{j}-n$ sites by cosolvent molecules is given by the binomial distribution, $p\left(n,m_{j}\right)=\frac{m_{j}!}{n!(m_{j}-n)!}\phi_{j}^{n}{\left(1-\phi_{j}\right)}^{m_{j}-n}$. Hence, by referring to Equation (6), the molar concentration of the protein in the *j*-state dissolved in a binary solvent with all its $m_{j}$ first hydration shell sites occupied by water molecules is given by $C_{P_{j}{\left({W_{s}}_{j}\right)}_{m_{j}}}=C_{j}\phi_{j}^{m_{j}}$, where $C_{j}=\alpha_{j}^{-1}C_{P}\;x_{j}=\sum_{n=0}^{m_{j}}C_{P_{j}{\left({W_{s}}_{j}\right)}_{n}{\left({G_{s}}_{j}\right)}_{m_{j}-n}}$ is the total molar concentration of the protein in the *j*-state, independently on the occupation of the sites by water or cosolvent. Notice that $C_{P}=c_{}/M_{1}=n/N_{A}$ is the nominal molar concentration of monomers in solution. As a consequence, in a binary solvent, the effective equilibrium constant $K_{1j}$, which describes the transformation of a protein molecule by the 1-state to the *j*-state, irrespective of the composition of the first hydration shell, and the related *effective* Gibbs free energy change are

(10) $$\begin{array}{ccc} & & K_{1j}=\frac{{\left(\alpha_{j}^{-1}C_{P}\;x_{j}\right)}^{\alpha_{j}^{-1}}}{C_{P}\;x_{1}}=K_{W1j}\frac{\phi_{1}^{m_{1}}X_{W_{b}}^{\alpha_{j}^{-1}m_{j}-m_{1}}}{\phi_{j}^{\alpha_{j}^{-1}m_{j}}}=e^{-\Delta G_{1j}/\left(RT\right)} \end{array}$$

The composition of the system is expressed by the nominal molar fractions of water, $X_{W}$, cosolvent, $X_{G}$, and protein monomers, $X_{P}$, with the straightforward condition $X_{W}+X_{G}+X_{P}=1$. Consequently, the nominal composition of the solvent is

(11) $$\begin{array}{c} x_{G}=\frac{X_{G}}{1-X_{P}} \end{array}$$

Since $X_{P}$ and $x_{G}$ are fixed parameters characterizing the sample, in any conditions of protein distribution among the states and preferential solvation effects, the following two constraints should hold,

(12) $$\begin{array}{ccc} & & X_{W_{b}}=\left(1-X_{P}\right)\left(1-x_{G}\right)-X_{P}\sum_{j=1}^{N_{s}}m_{j}x_{j}\alpha_{j}^{-1}\phi_{j} \end{array}$$

(13) $$\begin{array}{ccc} & & X_{G_{b}}=\left(1-X_{P}\right)x_{G}-X_{P}\sum_{j=1}^{N_{s}}m_{j}x_{j}\alpha_{j}^{-1}\left(1-\phi_{j}\right) \end{array}$$

Notice that the effective parameters $K_{1j}$ and $\Delta G_{1j}$ can change with the composition (i.e., by varying $X_{P}$ or $x_{G}$), whereas the exact thermodynamic parameters $K_{W1j}$ and $\Delta G_{W1j}$ as well as $K_{\mathrm{ex}j}$ and $\Delta G_{\mathrm{ex}j}$, which refer to the two elementary processes of Equations (5) and (7), should be independent on $X_{P}$ and $x_{G}$. However, the Gibbs free energy change $\Delta G_{W1j}$ can be affected by pH and ionic strength, which could be modified by the presence of cosolvent molecules, if they possess acid-base or ionic properties (such as for some of the modified sugars exploited in this work; see Table S1). In order to deal with these cases, we separate an electrostatic term from all the other non-electrostatic terms [22,23], $\Delta G_{W1j}=\Delta G_{W,\mathrm{el},1j}+\Delta G_{W,\mathrm{nel},1j}$, and we write $\Delta G_{W,\mathrm{el},1j}=\alpha^{-1}G_{W,\mathrm{el},j}-G_{W,\mathrm{el},1}$ in the framework of the Debye–Hückel theory,

(14) $$\begin{array}{ccc} & & G_{W,\mathrm{el},j}=\frac{q_{e}^{2}Z_{j}^{2}}{8\pi \varepsilon_{0}\varepsilon R_{j}}\left(1-\frac{\kappa_{D}R_{j}}{1+\kappa_{D}\left(R_{j}+a\right)}\right) \end{array}$$

In this equation, $q_{e}=1.6\cdot 10^{-19}$ C is the charge of the proton, expressed in SI units, $\varepsilon_{0}$ is the vacuum permittivity, ε is the relative dielectric constant of the solvent, $Z_{j}$ is the number of the elementary charges provided by the *j*-protein, which is assumed to be a spherical macroion with radius $R_{j}$, and *a* is the average radius of the all the microions (including protein counterions) in solution. Of note, $Z_{j}$ can be simply calculated as a function of pH considering the side chain $pK_{a}$ values of the amino acids [24]. The reciprocal Debye–Hückel screening length, $\kappa_{D}={\left(2N_{A}q_{e}^{2}I/\left(\varepsilon_{0}\varepsilon k_{B}T\right)\right)}^{1/2}$ is an other parameter of $G_{W,\mathrm{el},j}$ ($k_{B}$ is Boltzmann’s constant). It depends on the ionic strength due to the molar concentration $C_{i}$ and the charge number $z_{i}$ of all *i*-microions, $I=\frac{1}{2}\sum_{i}z_{i}^{2}C_{i}$. On the basis of the electroneutrality condition, the molar concentration of protein counterions (assumed for the sake of simplicity to have a charge $|z_{\mathrm{ci}}|=1$) should be $C_{\mathrm{ci}}=C_{P}\sum_{j=1}^{N_{s}}x_{j}\alpha_{j}^{-1}\left|Z_{j}\right|$. We can hence write $I=I_{S}+I_{\mathrm{ci}}$, where $I_{S}$ is the added ionic strength and $I_{\mathrm{ci}}$ is the one due to counterions. Of note, $I_{S}$ is calculated considering microions due to charged buffer molecules, if any, and microions provided by the cosolvent, in the case they are charged species. The non-electrostatic term $\Delta G_{W,\mathrm{nel},1j}$ includes all the other contributions to the thermodynamic stability of the *j*-protein state. Its temperature dependency, as well as the one of $\Delta G_{\mathrm{ex}j}$, is written according to classical thermodynamics, $\Delta G=\Delta G^{\circ }+\left(\Delta C_{p}-\Delta S^{\circ }\right)\left(T-T_{\circ }\right)-\Delta C_{p}T\log\left(T/T_{\circ }\right)$, where $\Delta G^{\circ }$ and $\Delta S^{\circ }$ are the changes of Gibbs free energy and entropy at the reference temperature $T_{\circ }=298.15$ K, respectively, and $\Delta C_{p}$ is the change of the heat capacity at constant pressure, here considered to be independent on temperature. On the other hand, due to thermal expansion, molar volumes are also affected by temperature. Regarding water, according with Ref. [25], the molecular volume can be described by the approximation $\nu_{W_{b}}=\nu_{W_{b}}^{\circ }e^{\alpha_{W}\left(T-T_{\circ }\right)+\frac{1}{2}\beta_{W}{\left(T-T_{\circ }\right)}^{2}}$, where the optimum values of the molar water volume at $T_{\circ }$, the thermal expansivity at $T_{\circ }$ and its first derivative are $\nu_{W_{b}}^{\circ }=0.018$ L, $\alpha_{w}=2.5\cdot 10^{-4}$ K${}^{-1}$ and $\beta_{w}=9.8\cdot 10^{-6}$ K${}^{-2}$, respectively [26]. For cosolvent and protein molar volumes, we adopt a simpler approximation, just in terms of molar volumes and thermal expansivities at $T_{\circ }$: $\nu_{G_{b}}=\nu_{G_{b}}^{\circ }e^{\alpha_{G}\left(T-T_{\circ }\right)}$ and $\nu_{P}=\nu_{P}^{\circ }e^{\alpha_{P}\left(T-T_{\circ }\right)}$. The nominal molar concentration of monomeric proteins (seen in Equation (10)) is

(15) $$\begin{array}{ccc} C_{P} & = & \frac{X_{P}}{<\;\nu \;>} \end{array}$$

(16) $$\begin{array}{ccc} <\;\nu \;> & = & \nu_{P}X_{P}+\left(\nu_{W_{b}}\left(1-x_{G}\right)+\nu_{G_{b}}x_{G}\right)\left(1-X_{P}\right)\\ & & +X_{P}\sum_{j=1}^{N_{s}}m_{j}x_{j}\alpha_{j}^{-1}\left(\left(\nu_{{W_{s}}_{j}}-\nu_{W_{b}}\right)\phi_{j}+\left(\nu_{{G_{s}}_{j}}-\nu_{G_{b}}\right)\left(1-\phi_{j}\right)\right) \end{array}$$

where the average molar volume <ν> is calculated as a function of the molar volume occupied by water and by cosolvent in the sites of the *j*-state of the protein, $\nu_{{W_{s}}_{j}}$, and $\nu_{{G_{s}}_{j}}$, respectively. In practice, only the former is considered to differ from the bulk value, since it has been widely demonstrated that hydration water has a more compact structure than bulk water [27,28]. Accordingly, we write $\nu_{{W_{s}}_{j}}=\nu_{W_{b}}/d_{j}$, where $d_{j}$ is the relative mass density of hydration water, with typical values comprised in the range 1÷1.15. By combining Equations (9), (12) and (13), it is straightforward to derive the cosolvent molar fraction of the bulk solvent as a function of both the fixed sample parameters, $X_{P}$ and $x_{G}$, and the parameters depending on the interplay of the equilibrium processes, the molar fraction $x_{j}$ of nominal protein monomers that are forming the *j*-state and the water occupation fraction $\phi_{j}$ of the first hydration shell of each *j*-state,

(17) $$\begin{array}{ccc} & & x_{G_{b}}=\frac{x_{G}\left(1-X_{P}\right)-X_{P}\sum_{j=1}^{N_{s}}m_{j}x_{j}\alpha_{j}^{-1}\left(1-\phi_{j}\right)}{1-X_{P}\left(1+\sum_{j=1}^{N_{s}}m_{j}x_{j}\alpha_{j}^{-1}\right)} \end{array}$$

The nonlinear system of $2N_{s}$ equations, which includes Equations (3) and (8) (with $j=1,N_{s}$) and Equation (10) (with $j=2,N_{s}$), in which the parameters $X_{W_{b}}$, $C_{P}$, <ν> and $x_{G_{b}}$ are obtained from Equations (12)–(17), respectively, contains the following $2N_{s}$ unknown variables: $x_{j}$ and $\phi_{j}$ (both with $j=1,N_{s}$).

The system is solved by a numerical iterative method as described in the Section S1 of the Supplementary Materials. In such a way, we have a method able to derive, from the thermodynamic parameters $\Delta G_{k}^{\circ }$, $\Delta S_{k}^{\circ }$ and $\Delta {C_{p}}_{k}$ that describe the two categories of elementary processes (non electrostatic contribution of protein state formation in water (Equation (5)) and water replacement of a cosolvent molecule over the surface of any protein state (Equation (7)) the fraction $x_{j}$ of nominal protein monomers distributed in the *j*-state and the fraction $\phi_{j}$ of the $m_{j}$ first hydration shell sites over the protein surface occupied by water. Additionally, we are able to calculate the cosolvent molar fraction of the bulk solvent, $x_{G_{b}}$, the effective constants $K_{1j}$ and the related Gibbs free energy change $\Delta G_{1j}$. All these parameters are obtained as a function of the nominal protein molar fraction $X_{P}$, the nominal binary solvent composition $x_{G}$, the pH, the added ionic strength $I_{S}$ and the temperature *T*.

#### 2.3.2. Determination of SLDs

The results from this thermodynamic scheme allow also to calculate the SLDs of bulk solvent and protein hydration shells. As widely discussed by Refs. [29,30], since the volume of the cosolvent molecule is much larger than the one of water, we have to consider that the cosolvent attached to the protein surface can in part occupy the hydration sites of the second hydration shell. As a consequence, preferential solvation effects will change the composition of a region in the vicinity of the protein surface, called local domain, which will encompass the hydration sites of both the first and the second shell. More in detail, the number of sites occupied by water and cosolvent in the first hydration shell (corresponding to the number of water and cosolvent molecules attached to the protein surface) are $N_{W,j,1}=m_{j}\phi_{j}$ and $N_{G,j,1}=m_{j}\left(1-\phi_{j}\right)$, respectively. Hence, the number of hydration sites of the second layer occupied by cosolvent molecules attached to the protein will be $k_{j}=m_{j}\left(1-\phi_{j}\right)\left(\nu_{{G_{s}}_{j}}-\nu_{{W_{s}}_{j}}\right)/\nu_{W_{b}}$. Indicating by $m_{j,2}$ the total number of hydration sites of the second layer, the ones that remain available to be occupied with the bulk solvent (with composition $x_{G_{b}}$) will be $m_{j,2}-k_{j}$. We can then calculate the number of water and cosolvent molecules that occupies the available sites of the second hydration shell, according to $N_{W,j,2}=\left(m_{j,2}-k_{j}\right)\left(1-x_{G_{b}}\right)/\left(1+x_{G_{b}}\left(\nu_{G_{b}}/\nu_{W_{b}}-1\right)\right)$ and $N_{G,j,2}=\left(m_{j,2}-k_{j}\right)x_{G_{b}}/\left(1+x_{G_{b}}\left(\nu_{G_{b}}/\nu_{W_{b}}-1\right)\right)$, respectively. On this basis, the cosolvent molar fraction of the local domain is

(18) $$\begin{array}{ccc} & & x_{{G_{\mathrm{ld}}}_{j}}=\frac{N_{G,j,1}+N_{G,j,2}}{N_{G,j,1}+N_{G,j,2}+N_{W,j,1}+N_{W,j,2}} \end{array}$$

and the local domain molar volumes of water and cosolvent are

(19) $$\begin{array}{ccc} & & \nu_{{W_{\mathrm{ld}}}_{j}}=\frac{N_{W,j,1}\nu_{{W_{s}}_{j}}+N_{W,j,2}\nu_{W_{b}}}{N_{W,j,1}+N_{W,j,2}} \end{array}$$

(20) $$\begin{array}{ccc} & & \nu_{{G_{\mathrm{ld}}}_{j}}=\frac{N_{G,j,1}\nu_{{G_{s}}_{j}}+N_{G,j,2}\nu_{G_{b}}}{N_{G,j,1}+N_{G,j,2}} \end{array}$$

Hence, the SLDs of bulk solvent and local domain are

(21) $$\begin{array}{ccc} & & \rho_{0}=\frac{x_{G_{b}}b_{G}+\left(1-x_{G_{b}}\right)b_{W}}{x_{G_{b}}\nu_{G_{b}}+\left(1-x_{G_{b}}\right)\nu_{W_{b}}} \end{array}$$

(22) $$\begin{array}{ccc} & & \rho_{\mathrm{ld},j}=\frac{x_{{G_{\mathrm{ld}}}_{j}}b_{G}+\left(1-x_{{G_{\mathrm{ld}}}_{j}}\right)b_{W}}{x_{{G_{\mathrm{ld}}}_{j}}\nu_{{G_{\mathrm{ld}}}_{j}}+\left(1-x_{{G_{\mathrm{ld}}}_{j}}\right)\nu_{{W_{\mathrm{ld}}}_{j}}} \end{array}$$

where $b_{W}=r_{e}N_{W,e}$ and $b_{G}=r_{e}N_{G,e}$ are the scattering lengths of water and cosolvent, with $N_{W,e}$ and $N_{G,e}$ being the corresponding number of electrons and $r_{e}=0.28\cdot 10^{-12}$ cm the classical radius of the electron. Considering the intrinsic low resolution of SAXS, also due to mobility effects over the protein surface, the calculation of both the form factors P(q) (Equation (2)) and $P^{\left(1\right)}\left(q\right)$ (Equation (4)) with SASMOL is performed by assigning to all the sites of the first and the second hydration shell (their numbers are $m_{j}$ and $m_{j,2}$, respectively) a unique SLD, corresponding to $\rho_{\mathrm{ld},j}$ (Equation (22)).

#### 2.3.3. Effective Protein–Protein Structure Factor

The protein–protein structure factor S(q) in the presence of a mixture of $N_{s}$ protein states is due to a complex interplay of the partial structure factors $S_{j_{1},j_{2}}\left(q\right)$ between any $j_{1},j_{2}$ pair of states weighted by their relative populations, which in turn depend on pair interaction potentials $u_{j_{1},j_{2}}\left(r\right)$. Here, according to Pedersen et al. [31], we adopt a simpler point of view by taking into account a unique *effective* radial interaction potential u(r) between two protein particles, irrespective of their state. This potential is described by the HSDY (Hard-Sphere Double-Yukawian) model, $u\left(r\right)=u_{\mathrm{HS}}\left(r\right)+u_{\mathrm{YC}}\left(r\right)+u_{\mathrm{YA}}\left(r\right)$, which combines a hard-sphere (HS) term,

(23) $$\begin{array}{ccc} u_{\mathrm{HS}}\left(r\right) & = & \left\{\begin{array}{cc} \infty & \;r<2R\\ 0 & \;r>2R \end{array}\right., \end{array}$$

and two Yukawian terms, described by the equation $u_{Yk}\left(r\right)={B_{1}}_{k}\mathrm{ex}p\left[-{B_{2}}_{k}\left(r-2R\right)\right]/r$. They are a screened Coulombian (C) repulsive term, with ${B_{1}}_{C}=4\pi Z^{2}q_{e}^{2}/\left(\varepsilon_{0}\varepsilon {\left(1+\kappa_{D}R\right)}^{2}\right)$ and ${B_{2}}_{C}=\kappa_{D}$, and an attractive (A) term, with ${B_{1}}_{A}=-2JR$ and ${B_{2}}_{A}=1/d$. In these equations, *R* is the average protein radius. It is calculated as an average of the protein radii $R_{j}$ of any state, according to $R=\left(1/<\;\alpha^{-1}\;>\right)\sum_{j=1}^{N_{s}}x_{j}\alpha_{j}^{-1}R_{j}$, where $<\;\alpha^{-1}\;>=\sum_{j=1}^{N_{s}}x_{j}\alpha_{j}^{-1}$. The average net number of elementary electric charges is calculated in a similar manner, $Z=\left(1/<\;\alpha^{-1}\;>\right)\sum_{j=1}^{N_{s}}x_{j}\alpha_{j}^{-1}Z_{j}$. The attractive term depends on two parameters, *J*, the energy when two proteins are at contact, (r=2R), and the scale length *d*. All attractive contributions, such as van der Waals forces, dipole-dipole or similar interactions are represented by $u_{\mathrm{YA}}\left(r\right)$. In the presence of cosolvent, which can provide variations of the surface properties of proteins, the values of *J* and *d* can change in a way that is not easily rationalized. Therefore, we have decided to leave the two parameters free to change for each experimental condition investigated by SAXS. The calculus of S(q) on the basis of u(r) was carried out by using the perturbation of the Percus–Yevick (PY) structure factor, $S_{0}\left(q\right)$, due to the two Yukawian terms, on the basis of the Random-Phase Approximation (RPA) [32,33,34]. The details are shown in Section S3 of the Supplementary Materials.

#### 2.3.4. Global-Fit of SAXS Data

On the basis of the model described in the previous sections, we are able to set a unique fit of a batch of $N_{c}$ SAXS curves recorded for water solutions of the protein of interest (which can show different states) by varying protein concentration and in the presence of different amounts of a cosolvent. This so-called global-fit can include several series of SAXS measurements performed with distinct types of cosolvents, provided single samples never contain two or more types of cosolvents. More specifically, SAXS curves are labeled with $N_{p}=4$ *curve parameters*: protein *w*/*v* concentration at $T_{\circ }$, ${c_{}}^{\circ }$, temperature, *T*, type of cosolvent, G, and its concentration at $T_{\circ }$, $C_{G}^{\circ }$. The task is accomplished by minimising the merit function $H=\chi^{2}+\gamma \;L$, where $\chi^{2}$ is the average reduced chi-square

(24) $$\begin{array}{ccc} \chi^{2} & = & \frac{1}{N_{c}}\sum_{k=1}^{N_{c}}\frac{1}{N_{q,k}}\sum_{i=1}^{N_{q,k}}{\left(\frac{{\frac{d\Sigma }{d\Omega }}_{k,\mathrm{expt}}\left(q_{i}\right)-{\frac{d\Sigma }{d\Omega }}_{k,\mathrm{theo}}\left(q_{i}\right)}{\sigma_{k}\left(q_{i}\right)}\right)}^{2} \end{array}$$

In this equation, ${\frac{d\Sigma }{d\Omega }}_{k,\mathrm{expt}}\left(q_{i}\right)$ is the $k^{\mathrm{th}}$ measured SAXS curve recorded over a number $N_{q,k}$ of *q*-points, ${\frac{d\Sigma }{d\Omega }}_{k,\mathrm{theo}}\left(q_{i}\right)$ is the theoretical curve calculated on the basis of Equation (1) and $\sigma_{k}\left(q_{i}\right)$ is the experimental standard deviation. The other term of the merit function, *L*, is the regularization factor,

(25) $$\begin{array}{ccc} L & = & \sum_{i=1}^{2}\sum_{k=1}^{N_{c}}\sum_{p=1}^{N_{p}}{\left(1-\frac{X_{i,k^{\prime }}}{X_{i,k}}\right)}^{2}, \end{array}$$

which increases with the difference between the $i^{\mathrm{th}}$ single curve fitting parameter (i=1,2 refers to *J* and *d*, respectively) of the *k*-curve, $X_{i,k}$, and the one of the $k^{\prime }$-curve, $X_{i,k^{\prime }}$, where $k^{\prime }$ is the label of the curve having the same *curve parameters* of the *k*-curve but the $p^{\mathrm{th}}$ ($p=1,N_{p}$ refers to ${c_{}}^{\circ }$, *T*, G and $C_{G}^{\circ }$). The constant γ is selected in order to guarantee that when $\chi^{2}\approx 1$, indicating a good fit, the product γL is ≈10% of the merit function H. The present model has been included in the freely available GENFIT software [35].

#### 2.3.5. Myoglobin

According to a number of experimental as well as computational evidences [36,37,38,39,40,41,42,43], myoglobin (MB) in solution at pH=5.0 and as a function of temperature can be present in three states, native (*N*), intermediate (*I*), and unfolded (*U*). The native state is monomeric and its form factor has been calculated on the basis of the PDB entry 1wla [44]. The corresponding form factor has been then calculated with SASMOL. The average numbers of hydration sites in the first and in the second shell are found to be $m_{N}=404$ and $m_{N,2}=465$, respectively. The intermediate state is considered to be a compact dimer [42], which has proven to maintain its active form [36,38]. Its form factor has been calculated with SASMOL from the PDB entry 3vm9 [38]. The number of hydration sites are $m_{I}=753$ and $m_{I,2}=827$. The unfolded state of MB has been described by a set of 50 conformations obtained by FOX, a home made software that preserves the secondary structure of a native structure and randomly modifies the Ramachandran angles of the residues that do not belong to helices or strands [45]. Steric clashes are avoided by controlling the overlap between the van der Waal spheres associated to each atom. The input PDB entry 1wla has been adopted. The average form factor has been then calculated with SASMOL. The number of hydration sites in the first and in the second shell are $m_{U}=844$ and $m_{U,2}=1141$, respectively. The form factors of the $N_{s}=3$ states of MB are shown in the Figure S1 of the Supplementary Materials in the form of semi-logarithmic and Kratky plots, together with the coupling function β(q). The number of elementary charges for the *N*-state, $Z_{N}$, calculated on the basis of the primary sequence of MB and as a function of pH are reported in Table S1 of the Supplementary Materials. For the *I* and *N* states, we simply fixed $Z_{I}=2Z_{N}$ and $Z_{U}=Z_{N}$.

#### 2.3.6. Insulin

Insulin (IN) in water solution has been found to mainly form $N_{s}=4$ folded states, corresponding to monomers (1), dimers (2), tetramers (4) and hexamers (6) [46,47,48,49,50,51,52,53]. Monomers are formed by two polypeptide chains, named A and B, linked by two disulfide bridges. It is known that insulin is present in its hexameric form, which is the best way to store and stabilize the functional monomers. Once hexamers dissociate into monomers, dimers, and tetramers, they can be transported in the bloodstream and they are ready to exert their physiological activity  [54]. The basic processes of oligomers’ formation have been identified as follows [46],

(26) $$\begin{array}{c} \begin{array}{c} 2\;I_{1}⇌I_{2}\\ 2\;I_{2}⇌I_{4}\\ I_{2}+I_{4}⇌I_{6} \end{array} \end{array}$$

Related thermodynamic constants have been determined at room temperature. They are ${\bar{K}}_{12}=2.22\;10^{5}$ M${}^{-1}$, ${\bar{K}}_{24}=40$ M${}^{-1}$ and ${\bar{K}}_{46}=220$ M${}^{-1}$ [46,47]. These constants are connected to the effective constants defined in Equation (10) by the following relationships: ${\bar{K}}_{12}=K_{12}^{2}$, ${\bar{K}}_{24}={\left(K_{14}/K_{12}\right)}^{4}$ and ${\bar{K}}_{46}=K_{16}^{6}/\left(K_{14}^{4}K_{12}^{2}\right)$. Form factors of the different states have been calculated with SASMOL on the basis of the PDB entry 3aiy [55]. For the monomer, only chains A and B have been considered, for the dimer the chains A–D, for the tetramer the chains A–H and for the hexamer the whole PDB file (A-L chains). Figure S2 of the Supplementary Materials reports semi-logarithmic and Kratky plots of the form factors of the $N_{s}=4$ states and their coupling function β(q). For the monomer, the average numbers of hydration sites in the first and in the second shell are found to be $m_{1}=199$ and $m_{1,2}=268$, respectively. For the dimer, the tetramer and the hexamer corresponding values are: $m_{2}=320$, $m_{2,2}=385$; $m_{4}=548$, $m_{4,2}=625$; $m_{6}=741$, $m_{6,2}=739$. In Table S1 of the Supplementary Materials the number of elementary charges for the 1-state, $Z_{1}$, which has been obtained considering the primary sequence of IN and the pH of the solution are reported. For the other states we have simply fixed $Z_{j}=\alpha_{j}Z_{1}$.

## 3. Results

### 3.1. Myoglobin

**Figure 3:**
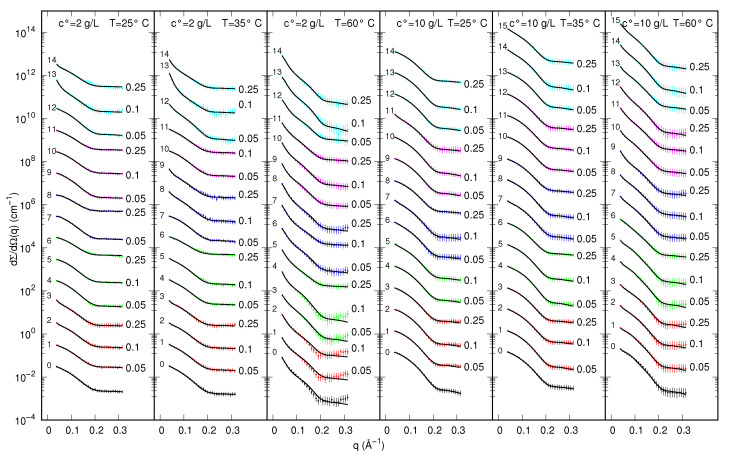
Experimental SAXS curves of MB in 10 mM phosphate buffer (pH=5) with and without ExtremoChem modified sugar superimposed with the best fits obtained with GENFIT (solid lines). Colors refer to the following conditions: no-modified sugar (black), EC312 (red), EC101 (green), EC311 (blue), EC202 (magenta), EC212 (cyan). Whenever present, the modified sugar concentration is reported on the right side of each curve in molar unit. Each column refers to a fixed temperature and MB concentration, as indicated on the top. Curves are multiplied by the factor $10^{k}$, with *k* being reported on the top right of each curve. Experimental standard deviations are reported as error bars every 10 points, for clarity.

SAXS curves recorded at the Elettra synchrotron (Austrian SAXS beam-line) for samples of myoglobin in the presence of five different ExtremoChem modified sugars, by varying protein or modified sugar concentration as well as temperature, are shown in Figure 3 in the form of semi-logarithm plots. To note, several curves show an upward curvature at low *q*, suggesting a predominant long-range attraction among the protein particles. Kratky plots, shown in Figure S3 of the Supplementary Materials, reveal, on the one hand, the presence of a main peak at a *q*-position that changes as a function of sample composition and temperature and, on the other hand, the absence of an asymptotic behavior at high *q*. These features suggest that the aggregation number of myoglobin can change with sample compositions and that most of the protein states are compact, also at the highest temperatures. Hence, since the information content of the SAXS dataset on the *U*-state is low, the ensemble of unfolded conformations calculated with the FOX method [45] has been left fixed. On the basis of these preliminary observations, the whole set of $N_{c}=92$ SAXS curves has been globally analyzed by using the new method introduced in Section 2.3. Three possible myoglobin states have been taken into account: two of them, the native monomer (*N*) and the intermediate dimer (*I*), are biologically active states, whereas the monomeric unfolded (*U*) state represents a denatured (inactive) form of the protein. The full list of the model parameters, together with their short descriptions and the validity range we have defined is reported in Table S2 of the Supplementary Materials. Best fitting curves are reported as solid lines in Figure 3: the high quality of the fit, in the entire *q*-range, can be appreciated. By fixing the dimensionless regularization parameter $\gamma =10^{-7}$, the overall merit function H=1.15 has been obtained, corresponding to $\chi^{2}=1.03$ (γL=0.12; see Equation (25)). The thermodynamic fitting parameters obtained by the simultaneous analysis of the whole set of SAXS data are reported in Table 1.

First of all, the results indicate that, in water at pH=5, the NI transition of myoglobin from native monomer to intermediate dimer has a low non-electrostatic reference Gibbs free energy barrier ($\Delta G_{W,\mathrm{nel},NI}^{\circ }=\left(2.95\pm 0.03\right)$ kJ mol${}^{-1}$, Table 1) when compared to the value related to the NU transition from native monomer to unfolded monomers ($\Delta G_{W,\mathrm{nel},NU}^{\circ }=\left(167\pm 3\right)$ kJ mol${}^{-1}$). Another fitting parameter, which is relevant in determining the effect of temperature, is the reference entropy variation, $\Delta S_{Wj_{1}j_{2}}^{\circ }$, related to the two NI and NU processes. The NI transition from native monomer to intermediate and still active dimer causes an increase in reference entropy, an effect that can be explained considering that this process determines the release of hydration water to the bulk solution. Indeed, since there are 404 hydration water molecules in the monomeric *N*-state and 753 in the dimeric *I*-state (see Section 2.3.5), 28 molecules of water for each monomer are released in solution when the dimer is formed, resulting in an increase in the reference entropy up to (564±6) J mol${}^{-1}$ K${}^{-1}$ (Table 1). On the other hand, when monomeric myoglobin switches from native to unfolded state (NU transition), a different scenario emerges. Although in the *U*-state the protein shell is surrounded by 844 water molecules, a value much higher if compared to 404 molecules that encircle the *N*-state, with a concomitant decrease in entropy, the formation of an unfolded disordered state is surely accompanied by a huge increase in entropy, so that the balance between the two phenomena leads to the observed large and positive value of the reference entropy change $\Delta S_{WNU}^{\circ }=\left(1600\pm 500\right)$ J mol${}^{-1}$ K${}^{-1}$, Table 1. The almost zero variation of the $\Delta {C_{p}}_{WNI}$ and the large and positive value $\Delta {C_{p}}_{WNU}=\left(8400\pm 400\right)$ J mol${}^{-1}$ K${}^{-1}$ are expected, considering the large accessible surface area of the protein unfolded state [56]. The other fitting parameters reported in Table 1 regard the changes of reference Gibbs free energy, reference entropy and heat capacity at constant pressure that occur when a modified sugar molecule bound to the myoglobin surface in each of the three envisaged *j*-states (*N*, *I* and *U*) is replaced by a water molecule. In general, we observe that whereas the experimental uncertainty of $\Delta G_{\mathrm{ex}j}^{\circ }$ is low (on average in the order of 1%), the ones of $\Delta S_{\mathrm{ex}j}^{\circ }$ and $\Delta {C_{p}}_{\mathrm{ex}j}$ are much larger, a result that can be in part explained considering that we have investigated our samples only at three different temperatures. These high uncertainties reflect the correctness of the global-fit SAXS data analysis method (see details in Section S2 of the Supplementary Materials), which does not lead to an overestimation of the parameters when their information content in the dataset is low.

**Figure 4:**
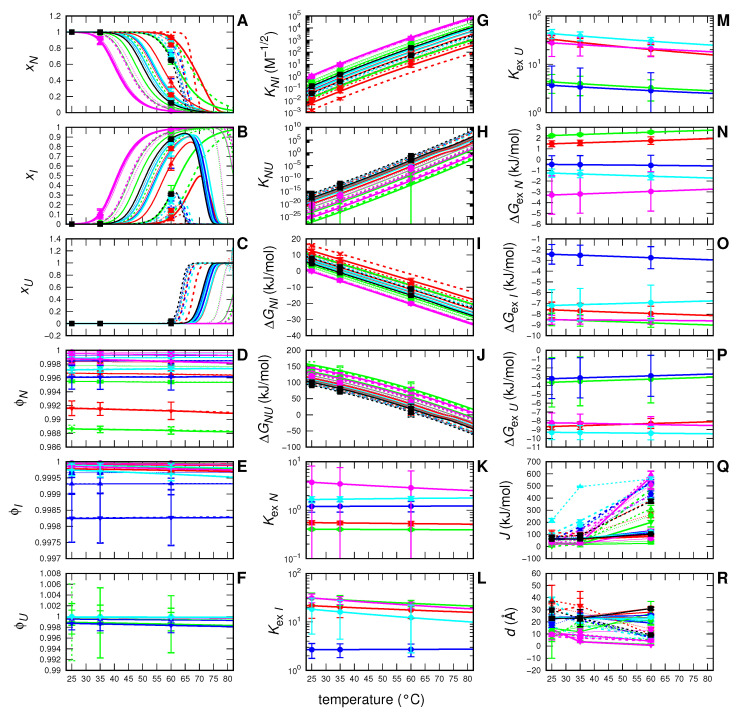
Temperature behaviors of the most relevant physical-chemical parameters (panels **A**–**R**) obtained by the global-fit of MB SAXS curves shown in Figure 3. Color refers to: no-modified sugar (black), EC312 (red), EC101 (green), EC311 (blue), EC202 (magenta), and EC212 (cyan). Thickness refers to: 0.05 M (thin), 0.10 M (intermediate), and 0.25 M (thick). Point-type refers to: no-modified sugar (square), 0.05 M (circle), 0.10 M (up-sided triangle), and 0.25 M (down-sided triangle). Dotted and solid lines refers to $c_{}^{\circ }=2$ g/L and $c_{}^{\circ }=10$ g/L, respectively.

The meaning of all the fitting parameters shown in Table 1 can be better appreciated by considering the temperature dependency of the most relevant physical-chemical parameters inherent to the adopted model that have been derived by them. Their trends are shown in Figure 4. Notice that in this figure the colors and the thickness (together with the symbols) of the curves have been assigned according to the type and the concentration of the modified sugar, respectively, whereas dotted and solid lines refer to 2 or 10 g/L myoglobin concentration, respectivley. Panels **A**, **B** and **C** report the molar fractions of the nominal myoglobin monomers distributed into the three different *N*, *I* or *U* states ($x_{N}$, $x_{I}$ and $x_{U}$, respectivley). It is possible to appreciate that, even if at $25^{\;\circ }$C the *N* monomers are the only fraction present in solution, the temperature rise gradually affects the protein state, resulting in the decay of the monomeric *N*-state and the concomitant formation of dimeric *I*-state. It is known that the oxygen binding rate constant of myoglobin dimer is similar to that of the monomer, whereas the oxygen dissociation rate constant of the dimer is smaller than that of the monomer [38]. Hence, our results could provide suggestions concerning monomer–dimer function and role. However, the particular pH and buffer conditions which do not resemble in vivo conditions, suggest not to infer them by this experimental set-up. Of note, although in our experiment we did not reach temperatures higher than $60^{\;\circ }$C, the adopted model with the fitting parameters derived by the set of SAXS data allows to predict that at higher temperatures the population of the *U*-state grows at the expense of the *I*-state. The fractions $\phi_{j}$ of first hydration shell sites of the *j* protein state occupied by water are reported in panels **D**, **E** and **F**. Since in our samples the presence of water is dominant, values of $\phi_{j}$ are very close to 1, with small but detectable differences, depending on the modified sugar type. Such small differences, on the basis of Equation (10), are sufficient to describe the modified sugar-induced modification of the effective equilibrium constant $K_{Nj}$ describing transition from the *N*-state to the *j*-state (j=I,U): results are shown in Figure 4, panels **G** and **H**. Corresponding Gibbs free energy changes $\Delta G_{Nj}$, which comprise both the electrostatic and the non-electrostatic contributions, are shown as a function of *T* in panels **I** and **J**. Exchange modified sugar–water equilibrium constants $K_{\mathrm{ex}j}$ for each of the three *j*-states are reported in panels **K**, **L** and **M** and corresponding Gibbs free energy changes $\Delta G_{\mathrm{ex}j}$ are in panels **N**, **O** and **P**. Notice that both parameters do not depend on protein or modified sugar concentration, but only on modified sugar type. Finally, in panels **Q** and **R**, we report the depth of the attraction protein–protein potential *J* and its scale length *d*, which have been treated as single-curve fitting parameters.

Protein–protein structure factors S(q) (Equation (S1) of the Supplementary Materials), calculated with the fitting parameters and included in the fitted ${\frac{d\Sigma }{d\Omega }}_{k,\mathrm{expt}}\left(q_{i}\right)$ function (Equation (24)), are plotted in Figure S4 of the Supplementary Materials. Corresponding effective radial interaction potentials u(r) are reported in Figure S5 of the Supplementary Materials.

Other fitting parameters of the model are the relative densities of hydration water, $d_{j}$. We have found similar values for each of the $N_{s}=3$ MB states, with an average value of 1.07±0.02. The average radius $R_{j}$ of *N*, *I* and *U* states found by the global-fit are (17.0±0.2) Å, (26.7±0.3) Å and (43±2) Å, respectively. The ionic strength due to the buffer results (10.0±0.1) mM.

We discuss in the next paragraphs results obtained in the absence of modified sugars and in the presence of each of the five investigated modified sugars.

#### 3.1.1. Myoglobin without and with Modified Sugar

In the absence of modified sugars, MB at 2 g/L maintains its monomeric form (*N*-state) up to about $60^{\;\circ }$C (Figure 4, panel **A**, black dotted lines), whereas the molar fraction of nominal MB monomers that are forming dimers (*I*-states) reaches the maximum peak of $x_{I}\approx 0.3$ (Figure 4, panel **B**, black dotted lines). At higher temperatures, dimers sharply disappear and the unfolded *U* state becomes the predominant species in solution (Figure 4, panel **C**, black dotted lines). On the contrary, at 10 g/L MB starts its transition from monomer to dimer at $45^{\;\circ }$C (Figure 4, panel **A**, black solid lines) and up to $65^{\;\circ }$C the molar fraction of nominal MB monomers that are forming dimers is as large as $x_{I}\approx 0.9$ (Figure 4, panel **B**, black lines).

The addition of the modified sugars produces different effects depending on the type of the compound used, but it is in general evident that when the cosolvents are used together with the highest concentration of protein, MB tends to have a marked transition from monomer to dimer and it becomes unfolded at temperature higher than $70^{\;\circ }$C. On the other hand, MB at 2 g/L shows a different behavior, leaving out the dimeric form, except for the two cosolvents that form ionic species in solution (EC101 and EC202).

#### 3.1.2. Myoglobin with EC312

Myoglobin at 2 g/L in the presence of EC312 maintains its native monomeric state with a slow transition to dimers at $\approx 60^{\;\circ }$C, which slightly depends on EC312 concentration (Figure 4, panel **A**, red lines). Dimers (*I*-state) do not overcome the fraction $x_{I}\approx 0.2$ of the myoglobin molecules in solution and gradually decrease and disappear at $65^{\;\circ }$C with the development of the unfolded state (Figure 4, panel **B**, red lines). On the contrary, myoglobin at 10 g/L in the presence of EC312 is prone to form dimers at $50^{\;\circ }$C when the EC312 concentration is 0.05 M, leading to a solution rich in dimers ($x_{I}\approx 0.9$) that unfold at $75^{\;\circ }$C. At increasing concentration of EC312, the NI transition shifts from $55^{\;\circ }$C to $70^{\;\circ }$C, twenty degrees more than what occurs to the protein without modified sugar. These results are also described by the behavior of the effective equilibrium constant $K_{NI}$ (Figure 4, panel **G**, red curves). For both 2 and 10 g/L MB concentrationa (dotted and solid red curves), $K_{NI}$, with an increasing concentration of EC312, is lower than the value without EC312 (black lines). This aspect underlines the tendency of MB with EC312 to maintain its monomeric *N*-state for temperatures higher than the protein without EC312. Moreover, the temperature increase leads to higher values of $K_{NI}$, corresponding to a a preference for the dimeric state. For MB in the monomeric *N*-state, the exchange constant $K_{\mathrm{ex}N}$ owns values lower than 1 (Figure 4, panel **K**, red line), indicating a preference to be surrounded by EC312. On the other hand, the dimeric and the unfolded states show an increase in $K_{\mathrm{ex}j}$ (panels **L** (j=I) and M (j=U)), suggesting the preference of these MB states to be solvated by water. The effect of EC312 in modifying protein–protein long-range interactions is not marked, as can be observed by comparing the structure factors S(q) shown in Figure S4 of the Supplementary Materials (red and black curves) and the corresponding u(r) reported in Figure S5 of the Supplementary Materials. Of note, at $60^{\;\circ }$C and 2 g/L myoglobin, a condition close to the NU transition, stronger attractive interactions among proteins have been seen, both with and without EC312, whereas at $60^{\;\circ }$C and 10 g/L, when most of the proteins are *I*-dimers, a less marked attraction is seen.

#### 3.1.3. Myoglobin with EC101

Depending on its concentration, EC101 strongly affects the transition of MB from native monomer to intermediate dimer (Figure 4, panels **A** and **B**, green lines). While, at lower modified sugar concentration, the decay of *N*-monomers in favor of *I*-dimers begins ≈$15^{\;\circ }$C earlier than for the samples without EC101 at both 2 or 10 g/L myoglobin, by increasing the EC101 concentration this transition occurs at higher temperatures. In particular, dimers begin to be present in solution at ≈$40^{\;\circ }$C and subsequently totally substitute the *N*-monomers. The unfolded state is not present, except at temperatures above $80^{\;\circ }$C and with lower concentration of modified sugar. The trends of the effective constant $K_{NI}$ (Figure 4, panel **G**) also confirms that by increasing EC101 concentration, especially at 10 g/L, the protein tends to remain in the monomeric *N*-state at higher temperatures than in the absence of EC101. Concerning the unfolded state, the very low values of $K_{NU}$ (Figure 4, panel **H**) show that there is no propensity for the protein to unfold except for temperatures higher than ≈$80^{\;\circ }$C and in presence of the lowest EC101 concentration. The exchange constant $K_{\mathrm{ex}j}$ varies according to the type of protein state. When MB is the *N* state, $K_{\mathrm{ex}N}$ is less than one (Figure 4, panel **K**, green line), showing its preference to be surrounded by modified sugar, while for the intermediate and the unfolded states (panels **L** and **M**, green lines), $K_{\mathrm{ex}j}$ is greater than one, underlining the preference of the protein in such states to be surrounded by water. Likewise the EC312 case, also EC101 shows weak effects in modifying protein–protein long-range interactions (Figures S4 and S5 of the Supplementary Materials, green and black curves), confirming the presence of more marked attractions at $60^{\;\circ }$C and 2 g/L myoglobin, which are weaker at $60^{\;\circ }$C and 10 g/L.

#### 3.1.4. Myoglobin with EC311

Myoglobin, at 2 and 10 g/L, in the presence of EC311, retains its monomeric *N*-state up to $55^{\;\circ }$C and $45^{\;\circ }$C, respectively (Figure 4, panels **A** and **B**, blue lines), similarly to the protein in the absence of EC311, showing only a slight dependence on the EC311 concentration. At 2 g/L and in the presence of EC311, myoglobin appears to be present mainly in the form of *N*-monomer, except for a small gap between $55^{\;\circ }$C and $70^{\;\circ }$C, in which a small amount of dimer starts to grow, but it does not exceed the fraction ≈0.3 of the particles in solution. At $70^{\;\circ }$C all dimers formed with myoglobin 2 g/L are unfolded, while, at MB 10 g/L, *I*-dimers’ fraction reach ≈0.9 and then disappear, with an increment of the unfolded state at $75^{\;\circ }$C. The equilibrium constant $K_{NI}$ (Figure 4, panel **G**, blue curves) slightly depends on EC311 concentration, which in turn resembles the one of protein in absence of EC311 (black lines). The results indicate that, with increasing quantities of EC311, the value of $K_{NI}$ decreases, highlighting a tendency of the protein to be present in its monomeric *N*-state at higher temperatures in respect to the protein in the absence of EC311. A similar behavior is found also during the transition NU: the low $K_{NU}$ values (Figure 4, panel **H**, blue curves) confirm the propensity of the protein, at low temperatures, to be present in the *N*-state until $60^{\;\circ }$C. The exchange constant $K_{\mathrm{ex}j}$ is close to 1 when the protein is present in the *N*-state (Figure 4, panel **K**, blue curve), suggesting that there is no preference to be surrounded by water or by EC311. On the other hand, when we consider the intermediate and the unfolded state, the $K_{\mathrm{ex}j}$ values rise slightly (Figure 4, panels **L** and **M**, blue curves), suggesting a preference of MB in these states to be surrounded by water. Additionally, for EC311, more pronounced effects on protein–protein long-range attractions (Figures S4 and S5 of the Supplementary Materials, blue curves) are seen at $60^{\;\circ }$C and 2 g/L myoglobin and moderate effects are seen both at $60^{\;\circ }$C and 10 g/L and at $35^{\;\circ }$C and 2 g/L.

#### 3.1.5. Myoglobin with EC202

In the presence of EC202, the behavior of MB at 2 and 10 g/L is quite similar (Figure 4, panels **A** and **B**, magenta lines), showing a bigger shift if compared to the protein without EC202, which increases additionally as a function of the EC202 concentration. This means that MB switches from *N*-monomer to dimer at lower temperatures with respect to the protein without EC202 (black lines). The transition occurs at around $40^{\;\circ }$C for MB 10 g/L, ten degrees before the normal transition temperatures of the protein without EC202. A bigger effect is evident for MB 2 g/L, when the protein, in presence of EC202, has the NI transition that occurs at $45^{\;\circ }$C, twenty degrees before the protein without modified sugar in solution. The unfolded fraction is almost absent, with a slight onset at the lowest modified sugar concentrations at around $80^{\;\circ }$C. The trends of $K_{NI}$ are almost independent on MB concentration (Figure 4, panel **G**, magenta lines) and only slightly dependent on EC202 concentration. The results confirm the tendency of the protein to be in the intermediate state at lower temperatures compared to what happens in absence of EC202. The transition from the native to unfolded state, on the other hand, is disadvantaged as the $K_{NU}$ value is almost constantly lower than 1, except for temperatures higher than $80^{\;\circ }$C (Figure 4, panel **H**, magenta lines). As in the EC312 case, also with EC202 there is a slight dependence on the modified sugar concentration, without any effect due to protein concentration. Indeed, curves of MB 2 and 10 g/L are almost superimposed. $K_{\mathrm{ex}j}$, which indicates the protein preference to be surrounded by water or modified sugar, is much greater than 1 in each of the three envisaged states. In particular, a slight decreasing trend of the $K_{\mathrm{ex}j}$ parameter can be noted as a function of temperature, which, however, is not considered very relevant. The modified sugar EC202 shows, in general, week protein–protein long-range attractions (Figures S4 and S5 of the Supplementary Materials, magenta curves), the most relevant occurring at $60^{\;\circ }$C both at 2 and 10 g/L myoglobin.

#### 3.1.6. Myoglobin with EC212

Myoglobin at 2 or 10 g/L, in the presence of EC212, retains the *N* state up to $55^{\;\circ }$C and $45^{\;\circ }$C, respectively, showing only a slight dependence on the EC212 concentration (Figure 4, panel **A** and **B**, cyan curves). While MB at 2 g/L does not show a fraction of dimers greater than $x_{I}\approx 0.3$, at 10 g/L MB, the dimers’ fraction reaches ≈0.9. In the first case (2 g/L MB), at around $70^{\;\circ }$C the dimer has completely disappeared, replaced by the unfolded state, while at 10 g/L, a similar behavior happens at temperatures above $75^{\;\circ }$C. Both $x_{N}$ and $x_{I}$ curves follow a trend that is very similar to that of protein in absence of modified sugars (black lines). The effective equilibrium constants $K_{NI}$ (Figure 4, panel **G**, cyan curves) are almost overlapping to the values in absence of EC212 (black lines). The exchange constant $K_{\mathrm{ex}j}$ is always greater than 1 for each of the considered states (*N*, *I* and *U*), highlighting a constant preference of the protein to be surrounded by water molecules.

### 3.2. Insulin

**Figure 5:**
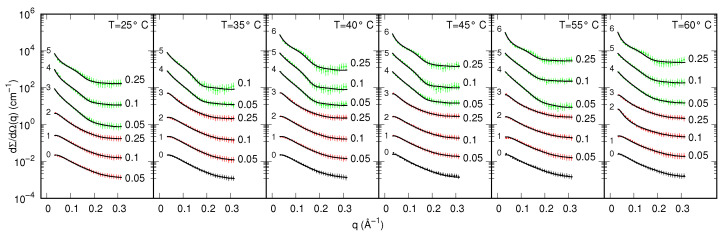
Experimental SAXS curves of 2 g/L IN in 10 mM phosphate buffer (pH=3) with and without ExtremoChem modified sugar superimposed with the best fits obtained with GENFIT (solid lines). Colors refer to the following conditions: no-modified sugar (black), EC312 (red), EC101 (green). Whenever present, the modified sugar concentration is reported on the right side of each curve in molar unit. Each column refers to a fixed temperature, as indicated on the top. Curves are multiplied by the factor $10^{k}$, with *k* being reported on the top right of each curve. Experimental standard deviations are reported as error bars every 10 points, for clarity.

SAXS curves recorded as a function of temperature for 2 g/L insulin in the presence of two modified sugars, EC312 and EC101, are shown in form of semi-logarithm plots in Figure 5. We first observe that several curves show an upward curvature at low *q*, indicating the prevalence of attraction forces at long range among the particles. In Figure S6 of the Supplementary Materials, Kratky plots of the experimental SAXS curves are shown: in all cases the presence of a main peak and the absence of asymptotic trends at high *q* revels the presence of compact IN shapes, with possible different aggregation states. On this basis, the simultaneous analysis of the $N_{c}=40$ SAXS curves shown in Figure 5 with the model introduced in Section 2.3 has been carried out by considering four possible states: monomer (1), dimer (2), tetramer (4), and hexamer (6). Table S3 of the Supplementary Materials reports the complete list of the model parameters, their description and the validity range.

Fitting curves, shown as solid lines in Figure 5, are well superimposed to the experimental curves in the entire *q*-range. The regularization parameter γ has been fixed to $10^{-7}$, leading to a merit function H=0.825 and a corresponding $\chi^{2}=0.764$ (γL=0.061, Equation (25)).

The main thermodynamic fitting parameters are reported in Table 2. We first observe that the non-electrostatic contribution of the reference Gibbs free energy changes, $\Delta {\bar{G}}_{W,\mathrm{nel},j_{1}j_{2}}^{\circ }$, related to the three processes shown in scheme (26), occurring in water at pH=3, are always negative, suggesting the presence of mechanisms other than charge-charge interactions that favor the formation of IN oligomers. We also notice that the reference entropy changes related to the three processes are positive, a results that can be understood considering the release of water molecules in the bulk when these oligomers are formed. Indeed, according to the number of waters sites found by SASMOL in the first hydration shell of the four species (Section 2.3.6), the numbers of water that are released due to the formation of dimers, tetramers or hexamers are are 78, 92, and 127, respectively. The heat capacities at constant pressure are found to be negative (Table 2) and affected by a quite large uncertainty (≈750 J mol${}^{-1}$ K${}^{-1}$). According to Ref. [57], negative values of heat capacity change are due to the fragility of hydrogen bonds between water molecules at the hydrophobic interfaces. However, of all the major thermodynamic variables measured for proteins, heat capacity is the one with the most different set of definitions and the richest set of implications for protein folding and binding. Its sign can distinguish apolar from polar solvation, and it imparts a temperature dependence to entropy and enthalpy that may change their signs and determine which of them will dominate [58]. The other thermodynamic parameters shown in Table 2 regards the modified sugar–water exchange in the surface of the four states of insulin that can be found in solution. The reference Gibbs free energy changes are obtained with low standard deviations (in the order of few percent), whereas larger uncertainties have been found for the reference entropy and heat capacity at constant pressure changes, confirming, such as for the MB case, that only a rough estimation of them can be derived from the SAXS dataset.

**Figure 6:**
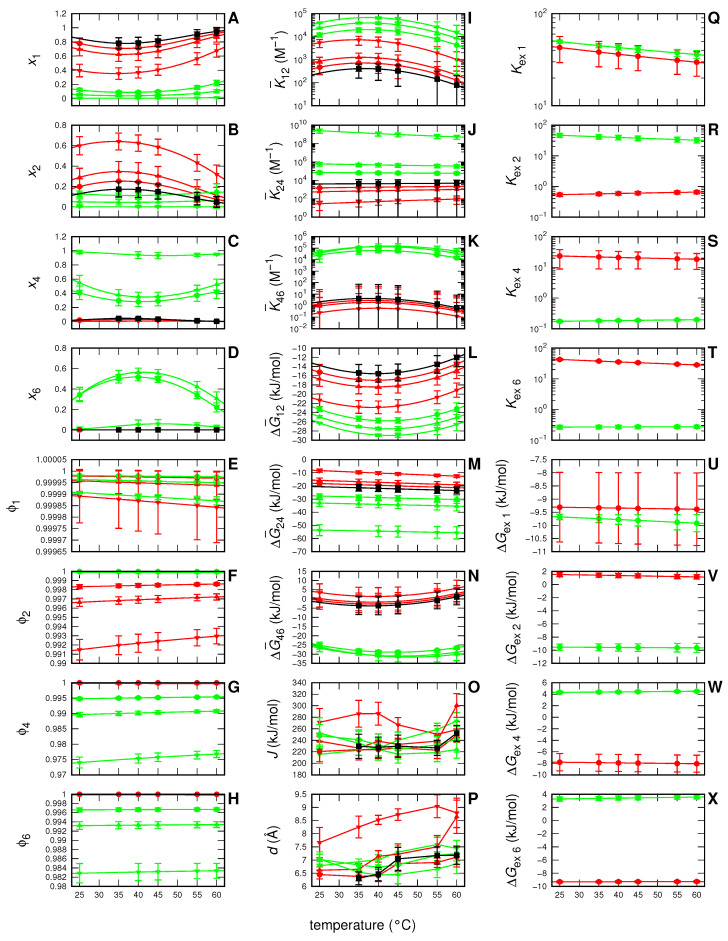
Temperature behaviors of the most relevant physical-chemical parameters (panels **A**–**X**) obtained by the global-fit of 2 g/L IN SAXS curves shown in Figure 5. Color refers to: no-modified sugar (black), EC312 (red), EC101 (green). Thickness refers to: 0.05 M (thin), 0.10 M (intermediate), 0.25 M (thick). Point-type refers to: no-modified sugar (square), 0.05 M (circle), 0.10 M (up-sided triangle), 0.25 M (down-sided triangle).

To fully understand the meaning of the fitting results, we report in Figure 6 the temperature behavior of all the physical-chemical parameters of the model derived by the fitting parameters. Of note, black curves refer to samples without modified sugar, whereas red and green curves are devoted to EC312 and EC101 compounds, respectively. In detail, panel **A**–**D** show the trends of the four fractions $x_{1}$, $x_{2}$, $x_{4}$ and $x_{6}$, respectivley. Panels **E**–**H** reports the fraction $\phi_{j}$ of first hydration shell occupied by water in the *j*-state (j=1,2,4,6). Effective equilibrium constants of the three processes reported in scheme (26) are shown in panels **I**–**K** and corresponding Gibbs free energy changes (including both non-electrostatic and electrostatic terms) in panels **L**–**N**. Regarding the modified sugar–water exchange processes, equilibrium constants and Gibbs free energy changes are reported in panels **Q**–**T** and **U**–**X**, respectively. Finally, panels **O** and **P** show the trend of the depth *J* and the scale length *d* of the long-range protein–protein attractive potential, which are free fitting parameters of each of the SAXS investigated curves.

These temperature trends firstly show that 2 g/L insulin molecules at pH=3, in the absence of modified sugars (black curves), are mainly present in monomeric or dimeric state, with a minimum $x_{1}\approx 0.8$ at $\approx 40^{\;\circ }$C and a maximum $x_{1}\approx 1$ at the highest temperatures.

The relative densities of hydration water have been found very similar for each of the $N_{s}=4$ IN states, with an average value of 1.06±0.01. Unique fitted values of the average radius $R_{j}$ of 1, 2, 4 and 6 state are (9.6±0.1) Å, (13.2±0.5) Å, (23.3±0.2) Å and (27.0±0.3) Å, respectively. The buffer contribution to the ionic strength is (4.1±0.2) mM.

The results of insulin without modified sugar and the effects provided by each of the two modified sugars are discussed in the next paragraphs.

#### 3.2.1. Insulin without and with Modified Sugar

Insulin in solution is mainly found in the form of a monomer. The molar fraction of nominal IN monomers that remain in the monomeric state in solution is indeed $x_{1}\approx 0.8$ (Figure 6, panel **A**, black line) while the rest are forming dimers ($x_{2}\approx 0.2$, Figure 6, panel **B**, black line) and neither tetramers nor hexamers are found (Figure 6, panels **C** and **D**, black lines). Both fractions $x_{1}$ and $x_{2}$ do not show a marked dependence on temperature, even if at around $60^{\;\circ }$C dimers disappears ($x_{2}$ tend to zero).

The addition of modified sugars, particularly one of them (EC101), induces a completely different behavior, with the prevalence of tetramers and hexamers that are negligible in the absence of the other modified sugars. Because hexamers represent the best oligomers to store and stabilize the functional monomers, this findings suggest that EC101 can be a successful compound for storing insulin.

#### 3.2.2. Insulin with EC312

The results show that the increase in the concentration of EC312 (red curves) determines a decrease in IN monomers in favor of dimers (Figure 6, panels **A** and **B**, red lines). The major difference is visible at 0.1 M and 0.25 M, when the fraction of dimers, $x_{2}$, increases from ≈0.3 to ≈0.6 with a slight dependence on the temperature until $45^{\;\circ }$C, after which monomers slowly increase up to $x_{1}\approx 0.8$. Tetramers and hexamers are not present in solution during the EC312 addition. This effect can be also observed in panels **I**–**K**: insulin in absence of modified sugars shows the lowest value of ${\bar{K}}_{12}$, indicating that the protein tends to stay in the monomeric state, whereas the addition of EC312 yields to higher ${\bar{K}}_{12}$ values and lower ${\bar{K}}_{24}$ and ${\bar{K}}_{46}$ values, confirming that EC312 favors the propensity of insulin to be found as a dimer in solution. The stabilization of the dimer in the presence of EC312 is clear considering the values of the exchange constants $K_{\mathrm{ex}j}$ reported in panels **Q**–**T**: the monomer, the tetramer and the hexamer shows $K_{\mathrm{ex}j}>1$, whereas for dimers $K_{\mathrm{ex}j}<1$, suggesting a preferential solvation of the dimer with EC312 in respect to water. We underline that, despite this preference only slightly modifies the water fraction in the first hydration shell of the dimers ($\phi_{2}$ has a minimum value of ≈0.992, panel **F**), this small effect is sufficient to provoke an important increase in the monomer–dimer effective equilibrium constant ${\bar{K}}_{12}$ (panel **I**). The trends of the IN–IN structure factors S(q), as well as the ones of the corresponding pair potentials u(r), reported in Figures S7 and S8 of the Supplementary Materials (red lines), clearly show a prevalence of long-range attractive forces in respect to repulsive forces. We also note that, up to 0.1 M EC312, the trends are quite similar to the ones observed for IN in the absence of modified sugar (black lines), without significant variations with *T*. Conversely, at 0.25 M EC312, the attractive interactions increase and become much more marked as the temperature increases.

#### 3.2.3. Insulin with EC101

EC101 behaves in a totally different way from EC312. Although the lowest concentration of EC101 retains a small fraction of monomers in solution ($x_{1}\approx 0.2$, Figure 6 panel **A**, green curves), which does not change considerably as a function of *T*, when insulin is mixed with EC101, insulin is mainly present as a tetramer or a hexamer. At 0.25 M EC101 only tetramers are in solution ($x_{4}\approx 1$), whereas at lower concentration, the EC101 causes the formation of hexamers ($x_{6}\approx 0.5–0.6$ at $40^{\;\circ }$C, panel **D**), with the remaining percentage occupied mainly by tetramers and in small part by monomers. The increase in temperature determines a negative slope of the $x_{6}$ *vs*. *T* curve, leading to a decrease in hexamers in favor of tetramers. The equilibrium constants ${\bar{K}}_{12}$, ${\bar{K}}_{24}$, and ${\bar{K}}_{46}$ (panels **I**–**K**, green curves) are bigger than the ones of IN in the absence of EC101 (black curves), and they grow additionally at increasing concentrations of EC101. Of note, the higher value of ${\bar{K}}_{24}$ (panel **J**, green curves), which describes how the equilibrium from dimers to tetramers changes in presence of EC101, confirms the prevalence of tetramer at 0.25 M EC101, as indicated in panel **C** (green curves). The exchange constants $K_{\mathrm{ex}j}$, reported in panels **Q**–**T** are found to be greater than 1 both for monomers and dimers and smaller than 1 for tetramers and hexamers. These results clearly show a preferential solvation of tetramers and hexamers with EC101 with respect to water and an opposite preference of monomers and dimers for water. This is the mechanism that shows the capability of EC101 in stabilizing tetramers and hexamers. Concerning the protein–protein structure factors and the related pair potentials of IN in the presence of EC101 (Figures S7 and S8 of the Supplementary Materials, green lines), the results show that the prevalence of long-range attractive forces at any EC101 concentration, which grow with temperature.

## 4. Discussion and Conclusions

We have shown that, by using an approach that includes both structural and thermodynamic features of a protein in solution, it is possible to extract from a batch of SAXS curves recorded at several conditions of temperature and protein as well as cosolvent concentrations crucial information regarding the stabilizing effects of cosolvents. The model we have developed focuses on the preferential water solvation properties over the surface of each of the distinct states that proteins can form in solution and shows how the modifications of these properties, due to the presence of a cosolvent, can provide changes in the distribution of protein molecules among the different states. Although SAXS experiments can only concern a limited number of conditions in terms of temperature and proteins or cosolvent concentration, most of the fitting parameters of our model do not refer to a specific experiment but to the whole set of thermodynamic laws that regulate the behavior of the protein system at any physical-chemical condition. An important consequence of this approach is the possibility to calculate the phase-diagram of the protein as a continuous function of temperature and cosolute concentration.

**Figure 7:**
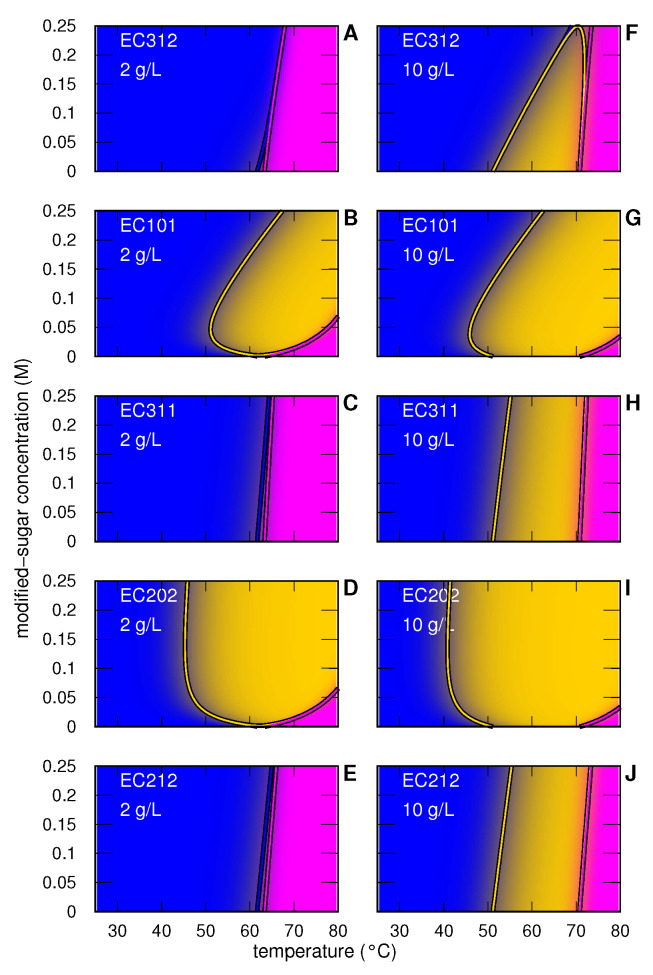
Temperature-modified sugar concentration phase-diagrams for MB in solution as obtained by the global-fit analysis of the SAXS curves. Panels in the same row refer to the same modified sugar, as indicated, whereas left and right column refer to 2 and 10 g/L MB concentration. The color code of each condition has been calculated by mixing, according to the protein *j*-state distribution ($x_{j}$), the following pure colors assigned to each *j*-state: *N* (blue), *I* (gold) and *U* (magenta). Solid lines are the contour levels corresponding to $x_{j}=0.5$ and their color has been assigned on the basis of the *j*-state. (Panels **A**–**J**) refer to the type of modified sugar and MB concentration as shown on the top left.

**Figure 8:**
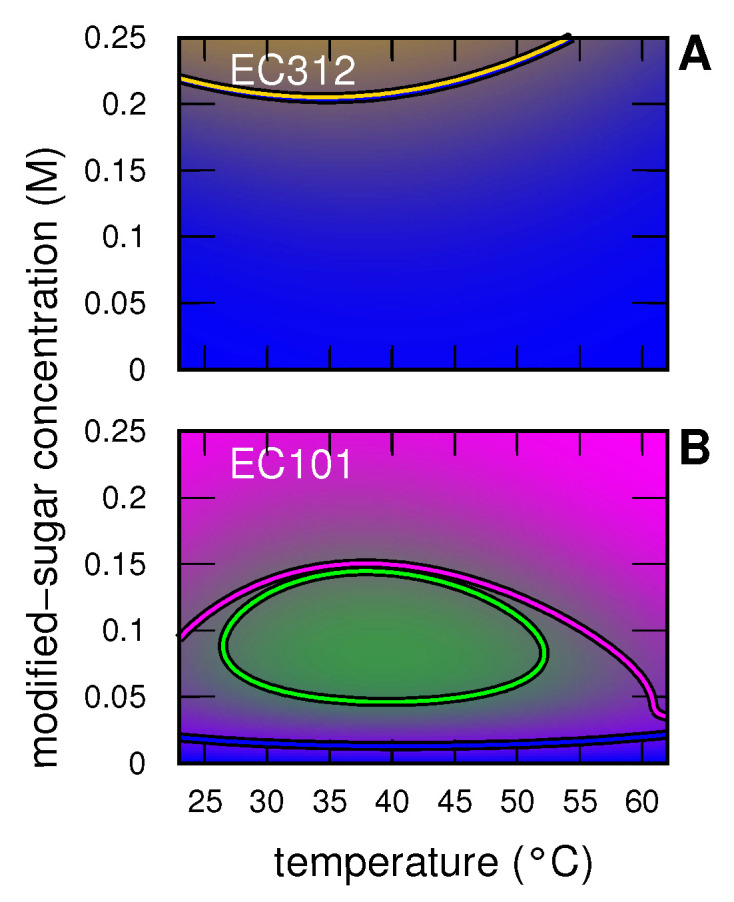
Temperature-modified sugar concentration phase-diagrams for 2 g/L IN in solution as obtained by the global-fit analysis of the SAXS. (Panels **A** and **B**) refer to EC312 and EC101, as indicated. The color code of each condition has been calculated by mixing, according to the protein *j*-state distribution ($x_{j}$), the following pure colors assigned to each *j*-state: monomers (j=1, blue), dimers (j=2, gold), tetramers (j=4, magenta) and hexamers (j=6, green). Solid lines are the contour levels corresponding to $x_{j}=0.5$ and their color has been assigned on the basis of the *j*-state.

Phase-diagrams derived by the two sets of SAXS data that we have analyzed in this work are shown in Figure 7, for MB in the presence of five ExtremoChem modified sugars, and in Figure 8 for IN in contact with two of these modified sugars. These diagrams contain the same information provided by the plots of $x_{j}$ shown in Figure 4 (panels **A**–**C**) and Figure 6 (panels **A**–**D**) but allow a more immediate visualization of the achieved results. Of note, the solid lines represent the thermodynamic condition in which at least one $x_{j}$ is 0.5. Regarding the MB case, Figure 7 (panels **A** and **F**) shows that EC312 is the best stabilizing modified sugar, since, at 0.25 M, it preserves the monomeric *N*-state (blue area) up to ≈$65^{\;\circ }$C. On the other hand, we see that 0.25 M EC101 (panels **B** and **G**) stabilize the *N*-state as well as the folded and active dimeric *I*-state (gold area) against the unfolded *U*-state (magenta area). EC311 (panels **C** and **H**) looks similar to EC312, but at 10 g/L MB it better stabilizes the *I*-state. We also see that EC202 (panels **D** and **I**) determines the largest stabilization area of the *I*-state against the *U*-state. Finally, the EC212 (panels **E** and **J**) results are similar to the EC312 ones, but with a more marked stabilization of the *I*-state at 10 g/L MB. In general, it is worth noting that the phase-diagrams of uncharged compounds (EC312, EC311 and EC212) are qualitatively similar and differ from the phase-diagrams of the two charged compounds (EC101 and EC202), which have an evident stabilization effect of the active *I*-state.

Regarding insulin, the phase-diagrams shown in Figure 8 confirm a totally different behavior in the presence of EC312 (panel **A**) with respect to EC101 (panel **B**): the former mainly stabilizes the monomer state, at least up to ≈0.2 M, the latter, at 0.25 M, promotes the tetramers, whereas at concentrations between 0.05 and 0.15 M and temperatures comprised between $25^{\circ }$ and $50^{\;\circ }$C favors the presence of hexamers.

Comparing the results obtained with MB and IN proteins, we could infer that the stabilizing effect of the tested compounds works as a specific binomial modified sugar protein. Although some sugars, such as trehalose, are commonly known to be stabilizers for biological macromolecules, their effect is always related to the specific protein. In particular, when dealing with proteins that present oligomeric equilibria, compounds efficiency in stabilizing each particular species is to be tested, since it depends on many features characterizing the macromolecule (charge, cavities, exposed groups, flexibility, etc.).

SAXS data also contain information regarding the long-range interactions of proteins, which our model is able to dissect. In the case of both MB at pH=5 and IN at pH=3 our result indicate the attractive forces dominate with respect to Coulumbian repulsion, in particular at the highest concentrations of modified sugar and temperature. Although our SAXS *q*-range does not allow to clearly identify the presence and the structure of high molecular weight species, our data suggest that they would be present, probably as unspecific aggregates. Further experimental evidences will be necessary to confirm this aspect.

The overall results achieved with the present study suggest that synchrotron-based SAXS technique, combined with advanced data analysis methods, is an invaluable tool for obtaining a detailed picture of thermal stability, oligomer distribution and long-range interactions of proteins in the presence of cosolvents.

## Figures and Tables

**Table 1:** Thermodynamic fitting parameters obtained by the global-fit of MB SAXS curves shown in Figure 3. $\Delta G_{W,\mathrm{nel},j_{1}j_{2}}^{\circ }$, $\Delta S_{Wj_{1}j_{2}}^{\circ }$ and $\Delta {C_{p}}_{Wj_{1}j_{2}}$: changes of non-electrostatic reference Gibbs free energy, reference entropy and heat capacity at constant pressure, respectively, occurring at the $j_{1}j_{2}$ transition; $\Delta G_{\mathrm{ex}j}^{\circ }$, $\Delta S_{\mathrm{ex}j}^{\circ }$ and $\Delta {C_{p}}_{\mathrm{ex}j}$: changes of reference Gibbs free energy, reference entropy and heat capacity at constant pressure, respectively, occurring at the modified sugar–water exchange over the *j*-state.

$j_{1}j_{2}$	$\Delta G_{W,\mathrm{nel},j_{1}j_{2}}^{\circ }$	$\Delta S_{Wj_{1}j_{2}}^{\circ }$	$\Delta {C_{p}}_{Wj_{1}j_{2}}$
	kJ mol${}^{-1}$	J mol${}^{-1}$ K${}^{-1}$	J mol${}^{-1}$ K${}^{-1}$
NI	2.95 ± 0.03	564 ± 6	0 ± 2
NU	167 ± 3	1600 ± 500	8400 ± 400
*j*	$\Delta G_{\mathrm{ex}j}^{\circ }$	$\Delta S_{\mathrm{ex}j}^{\circ }$	$\Delta {C_{p}}_{\mathrm{ex}j}$
	kJ mol${}^{-1}$	J mol${}^{-1}$ K${}^{-1}$	J mol${}^{-1}$ K${}^{-1}$
EC312			
*N*	1.5 ± 0.2	−10 ± 10	−7 ± 3
*I*	−8 ± 1	10 ± 10	−9 ± 6
*U*	−8.7 ± 0.5	−10 ± 9	2 ± 3
EC101			
*N*	2.2 ± 0.1	−9 ± 4	3 ± 3
*I*	−8.5 ± 0.7	9 ± 6	4 ± 7
*U*	−4 ± 1	−10 ± 7	0 ± 8
EC311			
*N*	−0.4 ± 0.6	2 ± 4	1 ± 5
*I*	−2.4 ± 0.8	9 ± 9	3 ± 7
*U*	−3 ± 4	−9 ± 2	−1 ± 4
EC202			
*N*	−3 ± 3	−10 ± 10	0 ± 4
*I*	−8.5 ± 0.5	1 ± 4	7 ± 6
*U*	−8 ± 1	6 ± 9	−7 ± 7
EC212			
*N*	−1.3 ± 0.4	9 ± 7	−7 ± 8
*I*	−7 ± 2	−8 ± 8	3 ± 3
*U*	−9.3 ± 0.5	3 ± 3	−2 ± 4

**Table 2:** Thermodynamic fitting parameters obtained by the global fit of IN SAXS curves shown in Figure 5. $\Delta {\bar{G}}_{W,\mathrm{nel},j_{1}j_{2}}^{\circ }$, $\Delta {\bar{S}}_{Wj_{1}j_{2}}^{\circ }$ and $\Delta {\bar{C_{p}}}_{Wj_{1}j_{2}}$: changes of non-electrostatic reference Gibbs free energy, reference entropy and heat capacity at constant pressure, respectively, occurring at the $j_{1}j_{2}$ transition (Equation (26)); $\Delta G_{\mathrm{ex}j}^{\circ }$, $\Delta S_{\mathrm{ex}j}^{\circ }$ and $\Delta {C_{p}}_{\mathrm{ex}j}$: changes of reference Gibbs free energy, reference entropy and heat capacity at constant pressure, respectively, occurring at the modified sugar–water exchange over the *j*-state.

$j_{1}j_{2}$	$\Delta {\bar{G}}_{W,\mathrm{nel},j_{1}j_{2}}^{\circ }$	$\Delta {\bar{S}}_{Wj_{1}j_{2}}^{\circ }$	$\Delta {\bar{C_{p}}}_{Wj_{1}j_{2}}$
	kJ mol${}^{-1}$	J mol${}^{-1}$ K${}^{-1}$	J mol${}^{-1}$ K${}^{-1}$
12	−25.4 ± 0.3	302 ± 3	−5200 ± 700
24	−16 ± 1	60 ± 10	−100 ± 800
46	−28.5 ± 0.3	396 ± 4	−6400 ± 700
*j*	$\Delta G_{\mathrm{ex}j}^{\circ }$	$\Delta S_{\mathrm{ex}j}^{\circ }$	$\Delta {C_{p}}_{\mathrm{ex}j}$
	kJ mol${}^{-1}$	J mol${}^{-1}$ K${}^{-1}$	J mol${}^{-1}$ K${}^{-1}$
EC312			
1	−9.3 ± 0.8	2 ± 5	0 ± 4
2	1.5 ± 0.3	10.0 ± 0.1	3 ± 5
4	−8 ± 2	8 ± 5	−5 ± 6
6	−9.3 ± 0.1	−1 ± 5	−2 ± 4
EC101			
1	−9.7 ± 0.1	6 ± 7	8 ± 9
2	−9.5 ± 0.4	4 ± 9	−9.5 ± 0.2
4	4.3 ± 0.2	−5 ± 2	−10 ± 2
6	3.3 ± 0.4	−8 ± 2	−9 ± 6

## Data Availability

The data presented in this study are available on request from the corresponding author.

## Supplementary Materials

The following supporting information can be downloaded at: https://www.mdpi.com/article/10.3390/life12010123/s1, Figure S1: Form factors of the $N_{s}=3$ states of MB; Figure S2: Form factors of the $N_{s}=4$ states of IN; Table S1: Experimental pH values determined as a function of the concentration of EC101 or EC202 modified sugar; Table S2: Overview of the model parameters and their validity range used in the global-fit analysis of $N_{c}=92$ SAXS curves of MB samples; Table S3: Overview of the model parameters and their validity range used in the global-fit analysis of $N_{c}=40$ SAXS curves of IN samples; Section S1: Numerical determination of $x_{j}$ and $\phi_{j}$; Section S2: Minimization of the merit function; Section S3: Calculation of S(q); Figure S3: Kratky plots of the experimental SAXS curves of MB; Figure S4: Protein-protein structure factors obtained by the analysis of SAXS data of MB; Figure S5: Protein-protein pair potentials obtained by the analysis of SAXS data of MB; Figure S6: Kratky plots of the experimental SAXS curves of 2 g/L IN; Figure S7: Protein-protein structure factors obtained by the analysis of SAXS data of IN; Figure S8: Protein-protein pair potentials obtained by the analysis of SAXS data of IN.
