# Supplementary material for: SAXS Reveals the Stabilization Effects of Modified Sugars on Model Proteins
Source: Life (Basel). 2022 Jan 15;12(1):123. doi: 10.3390/life12010123 (PMC8778440; doi:10.3390/life12010123)
Supplement: Supplementary file 1 [file life-12-00123-s001.zip › life-1529827-supplementary.pdf]

Supplementary Materials

SAXS Reveals the Stabilization Effects of Modified-Sugars on Model Proteins

Astra Piccinini [1], Eva C. Lourenço [2], Osvaldo S. Ascenso [2], Maria Rita Ventura [3], Heinz Amenitsch [4], Paolo Moretti [1], Paolo Mariani [1], Maria Grazia Ortore [1] and Francesco Spinozzi [1,*]

[1] Department of Life and Environmental Sciences, Polytechnic University of Marche, 60131 Ancona, Italy; a.piccinini@pm.univpm.it (A.P.); p.moretti@pm.univpm.it (P.M.); p.mariani@univpm.it (P.M.); m.g.ortore@univpm.it (M.G.O.)

[2] Extremochem, Rua Ivone Silva, 1050-124 Lisboa, Portugal; eva.lourenco@extremochem.com (E.C.L.); osvaldo.ascenso@extremochem.com (O.S.A.)

[3] Instituto de Tecnologia Química e Biológica António Xavier, Universidade Nova de Lisboa, 2780-157 Oeiras, Portugal; rita.ventura@extremochem.com

[4] Institute for Inorganic Chemistry, Graz University of Technology, 8010 Graz, Austria; amenitsch@tugraz.at

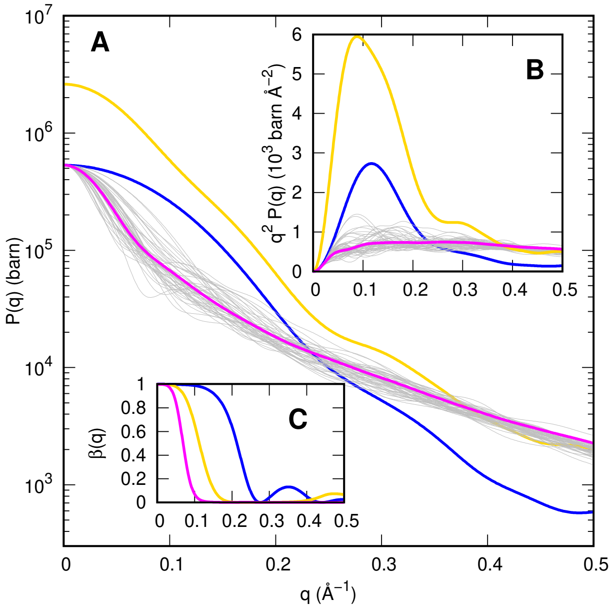

Figure S1: Form factors of the $N_s = 3$ states of MB calculated with the SASMOL method[1]. The blue curve represents the form factor of the native state (N) obtained on the basis of the PDB entry 1wla[2]. The gold curve is the form factor of the intermediate (I) dimeric state calculated from the PDB entry 3vm9[3]. Grey curves are the form factors of the 50 conformations obtained by FOX[4], whose average, corresponding to the the unfolded state (U), is represented by the magenta curve. Form factors are shown in the semi-logarithmic (panel **A**) and in the Kratky (panel **B**) plots. Coupling functions are reported in panel **C**.

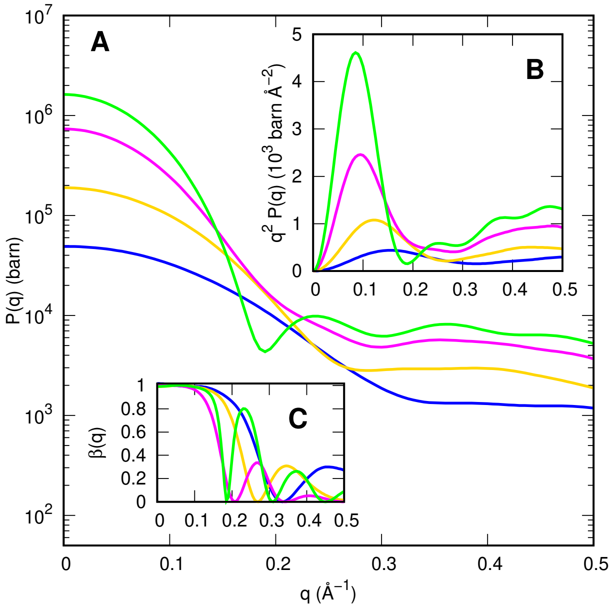

Figure S2: Form factors of the $N_s = 4$ states of IN calculated with the SASMOL method. Curves of monomer (1), dimer (2), tetramer (4) and hexamer (6) states, calculated from the PDB entry 3aiy[5], by selecting chains A-B, A-D, A-H and A-L, respectivley, are shown in blue, gold, magenta and green, respectivley. Form factors are shown in the semi-logarithmic (panel **A**) and in the Kratky (panel **B**) plots. Coupling functions are reported in panel **C**.

buffer pH	G	C_G M	pH	MB Z_N	IN Z_1
5.00	–	–	5.00	17.6	
5.00	EC101	0.05	5.15	16.8	
5.00	EC101	0.10	5.25	16.4	
5.00	EC101	0.25	5.35	15.9	
5.00	EC202	0.05	5.10	17.0	
5.00	EC202	0.10	5.20	16.6	
5.00	EC202	0.25	5.30	16.1	
3.00	–	–	3.00		4.7
3.00	EC101	0.05	3.80		2.8
3.00	EC101	0.10	4.15		1.9
3.00	EC101	0.25	4.50		1.0

Table S1: Experimental pH values determined as a function of the concentration of EC101 or EC202 modified-sugar dissolved in 10 mM phosphate buffer at pH = 3 or pH = 5 and corresponding number of elementary charges for the N-state of myoglobin and for the 1-state of insulin.

Symbol	Description	Unit	Min	Max	Ref.
α_{w}	water thermal expansivity	10^{-4} K^{-1}	**2.5**		[6]
β_{w}	first derivative of water thermal expansivity	10^{-6} K^{-2}	**9.8**		[6]
α_{P}	protein thermal expansivity	10^{-4} K^{-1}	**1.15**		[7]
α_{G}	modified-sugar thermal expansivity	10^{-4} K^{-1}	**3.9**		[8]
$\nu^{\circ}_{\mathrm{W_b}}$	bulk water molar volume at $T_{\circ}$	L	**0.018**		[6]
ν°_{P}	MB monomer molar volume at $T_{\circ}$	L	**12.95**		[1a]
$\nu^{\circ}_{\mathrm{G_b}}$	bulk modified-sugar molar volume at $T_{\circ}$	L	**0.12**		
I_0	ionic strength due to charged buffer molecules	mM	1	20	
$\Delta G^{\circ}_{\mathrm{W,nel},j_1j_2}$	non-electrostatic reference Gibbs free energy change at the j_1j_2 transition	kJ mol^{-1}	−200	200	
$\Delta S^{\circ}_{\mathrm{W}j_1j_2}$	reference entropy change at the j_1j_2 transition	J mol^{-1}	−200	200	
$\Delta C_{p\mathrm{W}j_1j_2}$	heat capacity at constant pressure change at the j_1j_2 transition	J mol^{-1}	−10000	10000	
$\Delta G^{\circ}_{\mathrm{ex}j}$	modified-sugar water exchange reference Gibbs free energy change over the j-state	kJ mol^{-1}	−10	10	
$\Delta S^{\circ}_{\mathrm{ex}j}$	modified-sugar water exchange reference entropy change over the j-state	J mol^{-1}	−10	10	
$\Delta C_{p_{\mathrm{ex}j}}$	modified-sugar water exchange heat capacity at constant pressure change over the j-state	J mol^{-1}	−10	10	
d_j	relative mass density of the j-state hydration water		0.9	1.15	[9]
R_N	average radius of N-MB state	Å	12	18	
R_I	average radius of I-MB state	Å	20	40	
R_U	average radius of U-MB state	Å	30	70	
Z_N	number of elementary charges of N-MB		see Table S1		
J	protein-protein contact energy	kJ mol^{-1}	0	1000	[10]
d	attractive potential scale length	Å	0.5	100	[10]

Table S2: Overview of the model parameters and their validity range used in the global-fit analysis of $N_c = 92$ SAXS curves of MB samples. Fixed parameters are shown in bold. [a] Value obtained with the SASMOL method. The total number of fitting parameters are 242, corresponding to ≈ 2.6 parameters per curve.

Symbol	Description	Unit	Min	Max	Ref.
α_w	water thermal expansivity	10^{-4} K^{-1}	**2.5**		[6]
β_w	first derivative of water thermal expansivity	10^{-6} K^{-2}	**9.8**		[6]
α_P	protein thermal expansivity	10^{-4} K^{-1}	**1.15**		[7]
α_G	modified-sugar thermal expansivity	10^{-4} K^{-1}	**3.9**		[8]
$\nu^\circ_{\mathrm{W_b}}$	bulk water molar volume at $T_\circ$	L	**0.018**		[6]
ν°_P	IN monomer molar volume at $T_\circ$	L	**4.32**		[1a]
$\nu^\circ_{\mathrm{G_b}}$	bulk modified-sugar molar volume at $T_\circ$	L	**0.12**		
I_0	ionic strength due to charged buffer molecules	mM	1	20	
$\Delta\bar{G}^\circ_{\mathrm{W,nel,12}}$	non-electrostatic reference Gibbs free energy change at the 12 transition (Eq. 26)	kJ mol^{-1}	−50	0	[11]
$\Delta\bar{G}^\circ_{\mathrm{W,nel,24}}$	non-electrostatic reference Gibbs free energy change at the 24 transition (Eq. 26)	kJ mol^{-1}	−30	30	[11]
$\Delta\bar{G}^\circ_{\mathrm{W,nel,46}}$	non-electrostatic reference Gibbs free energy change at the 46 transition (Eq. 26)	kJ mol^{-1}	−30	30	[11]
$\Delta\bar{S}^\circ_{\mathrm{W}j_1j_2}$	reference entropy change at the j_1j_2 transition (Eq. 26)	J mol^{-1}	−40	40	
$\Delta\bar{C}_{p\mathrm{W}j_1j_2}$	heat capacity at constant pressure change at the j_1j_2 transition (Eq. 26)	J mol^{-1}	−10000	10000	
$\Delta G^\circ_{\mathrm{ex}j}$	modified-sugar water exchange reference Gibbs free energy change over the j-state	kJ mol^{-1}	−10	10	
$\Delta S^\circ_{\mathrm{ex}j}$	modified-sugar water exchange reference entropy change over the j-state	J mol^{-1}	−10	10	
$\Delta C_{p_{\mathrm{ex}j}}$	modified-sugar water exchange heat capacity at constant pressure change over the j-state	J mol^{-1}	−10	10	
d_j	relative mass density of the j-state hydration water		0.9	1.15	[9]
R_1	average radius of 1-IN state	Å	10	20	
R_2	average radius of 2-IN state	Å	10	30	
R_4	average radius of 4-IN state	Å	15	35	
R_6	average radius of 6-IN state	Å	15	45	
Z_1	number of elementary charges of 1-IN		see Table S1		
J	protein-protein contact energy	kJ mol^{-1}	0	1000	[10]
d	attractive potential scale length	Å	0.5	100	[10]

Table S3: Overview of the model parameters and their validity range used in the global-fit analysis of $N_c = 40$ SAXS curves of IN samples. Fixed parameters are shown in bold. [a] Value obtained with the SASMOL method. The total number of fitting parameters are 122, corresponding to ≈ 3.0 parameters per curve.

S1 Numerical determination of x_j and ϕ_j

By assuming small values of $X_{\rm P}$ and $x_{\rm G}$, in a $0^{\rm th}$ approximation, on the basis of Eq. 12 and 17, we fix $X_{\rm W_b}^{(0)} \simeq (1 - X_{\rm P})(1 - x_{\rm G})$ and $x_{\rm G_b}^{(0)} \simeq x_{\rm G}(1 - X_{\rm P})$. We calculate the charge Z_j of any protein state and, by assuming in the $0^{\rm th}$ approximation $I_{\rm ci} \simeq 0$, we calculate the related electrostatic free energy $G_{{\rm W,el},j}$ from Eq. 14. Hence from Eq. 8 we derive a $0^{\rm th}$ approximation of ϕ_j according to $\phi_j^{(0)} \simeq K_{{\rm ex}j}(1-x_{\rm G_b}^{(0)})/(K_{{\rm ex}j}-(K_{{\rm ex}j}-1)x_{\rm G_b}^{(0)})$. Subsequently, by entering $\phi_j^{(0)}$ in Eqs. 15-16, we derive $<\nu>^{(0)}$ and $C_{\rm P}^{(0)}$. By substituting these values into Eq. 10, we derive a $0^{\rm th}$ approximation of $K_{1j}^{(0)}$ and of the fraction of j-proteins $x_j^{(0)} \simeq \alpha_j(K_{1j}^{(0)}x_1^{(0)})^{\alpha_j}(C_{\rm P}^{(0)})^{\alpha_j-1}$ (with $j = 2, N_s$), all depending on the unknown variable $x_1^{(0)}$. By entering the values of $x_j^{(0)}$ in Eq. 3, we obtain an equation with a unique variable $x_1^{(0)}$, comprised between 0 and 1, which can be numerically solved. Subsequently, by entering the values of $x_j^{(0)}$ in Eqs. 12-16, we derive a $1^{\rm st}$ approximation of the variables $X_{\rm W_b}^{(1)}$, $x_{\rm G_b}^{(1)}$, $<\nu>^{(1)}$, $C_{\rm P}^{(1)}$ and $I_{\rm ci}$. Hence, again from Eq. 8, we derive $1^{\rm st}$ approximations of $\phi_j^{(1)}$ and, from these ones, $1^{\rm st}$ approximations of $K_{1j}^{(1)}$ and, finally of $x_j^{(1)}$. The iteration is repeated until $|x_j^{(i)} - x_j^{(i-1)}| < \tau$ and $|\phi_j^{(i)} - \phi_j^{(i-1)}| < \tau$, where τ is a small value in the order of 10^{-10}.

S2 Minimization of the merit function

The merit function $\mathcal{H}$ is minimized by using two numerical methods, both integrated in the GENFIT software. The first method exploits a Simulated Annealing (SA) procedure. All the fitting parameters are randomly moved within their validity range: a move that decreases $\mathcal{H}$ ($\Delta\mathcal{H} \leq 0$) is always accepted, whereas a move that increases $\mathcal{H}$ is accepted with probability $e^{-\Delta\mathcal{H}/T^*}$, where T^* is a temperature-like parameter. The initial value of T^* is set to $\mathcal{H}_0/2$, where $\mathcal{H}_0$ is the value of the merit function corresponding to a first random choice of the fitting parameters. This high value of T^* ensures that the minimization starts by accepting most of the random moves, avoiding to get stuck in local minima. Every N_c random moves of the fitting parameters (referred to as a sub-run), T^* is decreased by a constant factor (i.e. the system is "cooled") so that the path along the space of the parameters is guided toward the global minimum of $\mathcal{H}$. We have chosen a total of $N_s = 50$ sub-runs formed by $N_c = 50$ random moves. The second method is based on the well-known simplex (SX) algorithm for solving numerically linear programming problems[12] and is applied to the set of fitting parameters that has been obtained by the SA method. The coupled SA-SX procedure is repeated for $N_I = 20$ independent iterations and, for each repetition, all the SAXS curves are

randomly sampled from a Gaussian centered around the experimental value $\frac{d\Sigma}{d\Omega}_{k,\mathrm{expt}}(q_i)$ with standard deviation $\sigma_k(q_i)$ [13]. In this manner, a robust determination of the mean value $<X>$ and the standard deviation σ_X of each fitting parameters is achieved.

S3 Calculation of $S(q)$

The modification of the PY structure factor $S_0(q)$ caused by the two Yukawian terms of the pair potential within the RPA approximation is

$$S(q) = \frac{S_0(q)}{1+\beta <n> S_0(q)[U_{\mathrm{YC}}(q)+U_{\mathrm{YA}}(q)]}, \quad \text{(S1)}$$

$$S_0(q) = \left\{1-\frac{12\eta[\eta(3-\eta^2)-2]}{(1-\eta)^4}\frac{j_1(2qR)}{2qR}\right\}^{-1}. \quad \text{(S2)}$$

These equations contain the protein volume fraction, $\eta = <n> \frac{4}{3}\pi R^3$, and the isotropic Fourier Transform of the Yukawa potential, which reads $U_{\mathrm{Y}k}(q) = 4\pi B_{1k}(B_{2k}\sin(2qR)+q\cos(2qR))/(q^3+qB_{2k}^2))$, with k = C and A and $\beta = 1/(k_BT)$. The average number density $<n>$ is calculated within the thermodynamic model according to $<n>=<\alpha^{-1}>n$, with $n = C_{\mathrm{P}}/N_A$. The function $j_1(x)$ is the 1st order spherical Bessel function.

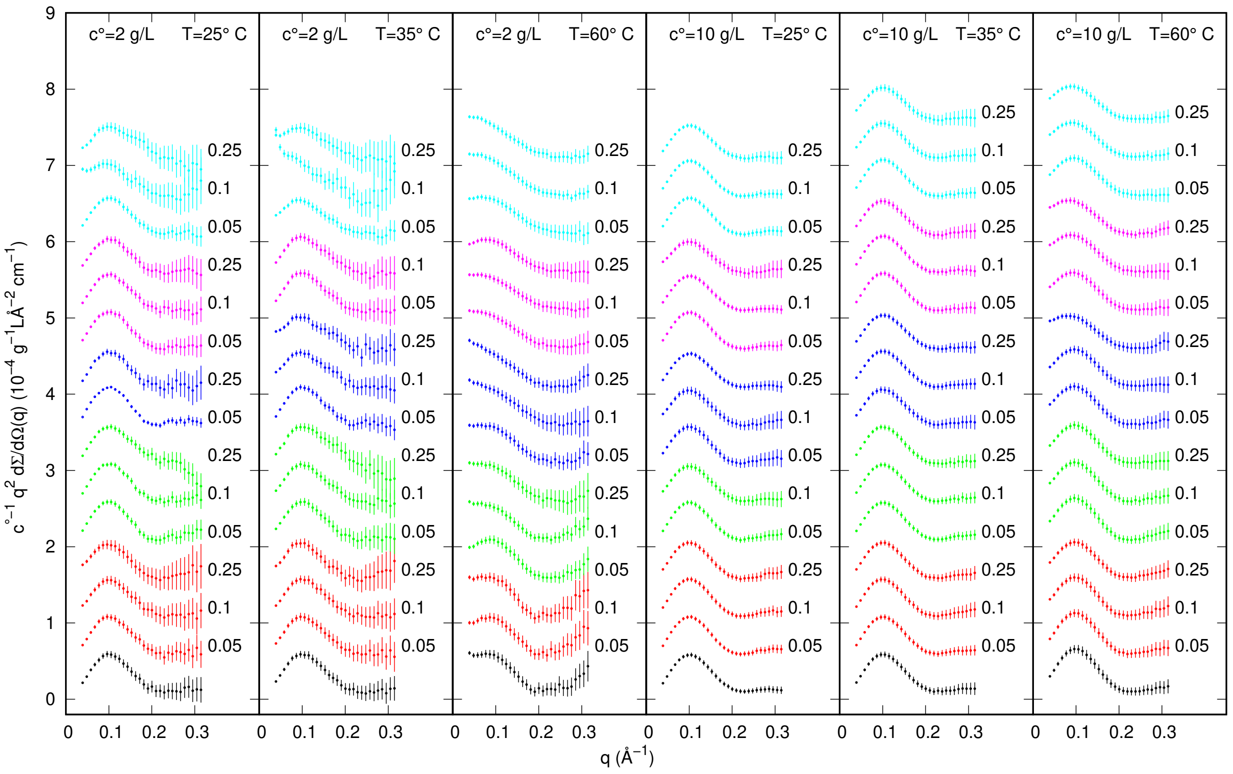

Figure S3: Kratky plots of the experimental SAXS curves of MB in 10 mM phosphate buffer (pH = 5) with and without ExtremoChem modified-sugar. Colors refer to the following conditions: no-modified-sugar (black), EC312 (red), EC101 (green), EC311 (blue), EC202 (magenta), EC212 (cyan). Whenever present, the modified-sugar concentration is reported on the right side of each curve in molar unit. Each column refers to a fixed temperature and MB concentration, as indicated on the top. Curves have been divided by the nominal w/v MB concentration ($c°$) and, in the same column, have been scaled by the factor 1 (in the unity of the y-axis). Experimental standard deviations are reported as error bars every 10 points, for clarity.

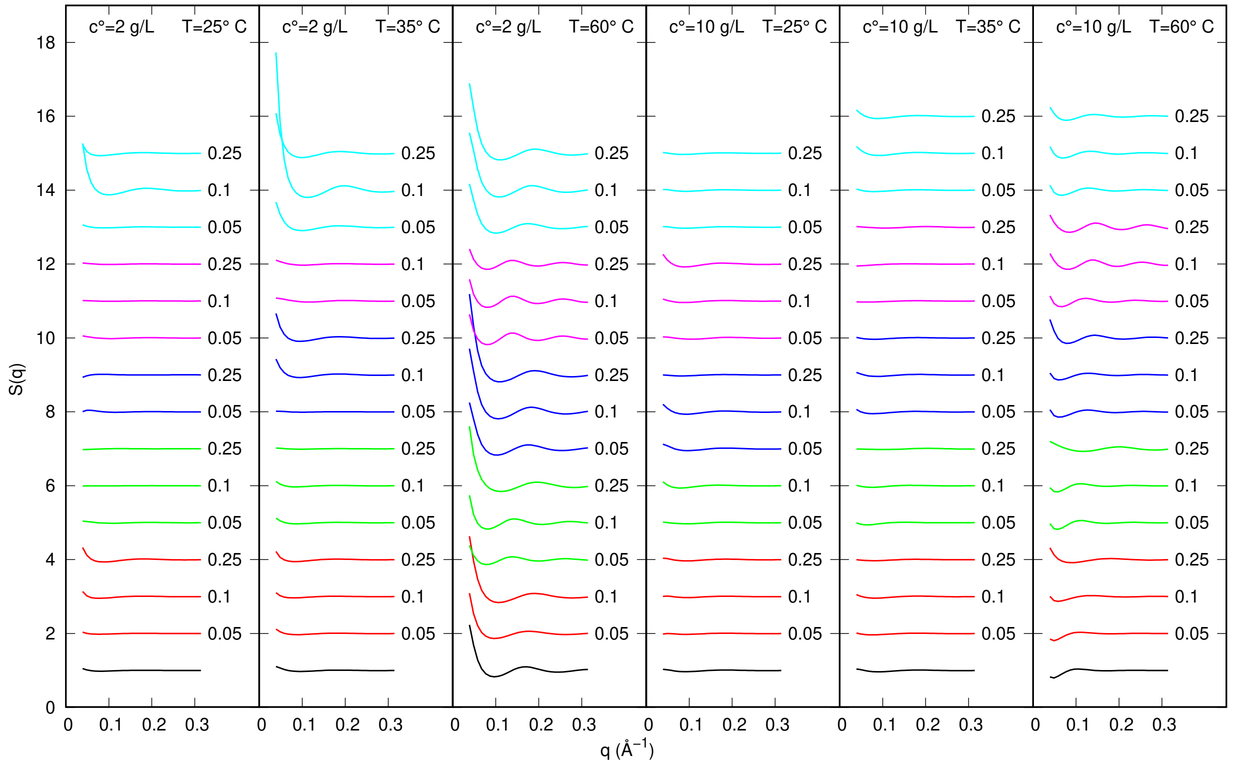

Figure S4: Protein-protein structure factors obtained by the analysis of SAXS data of MB samples shown in Figure 3. Curves are scaled by a factor 1 for clarity.

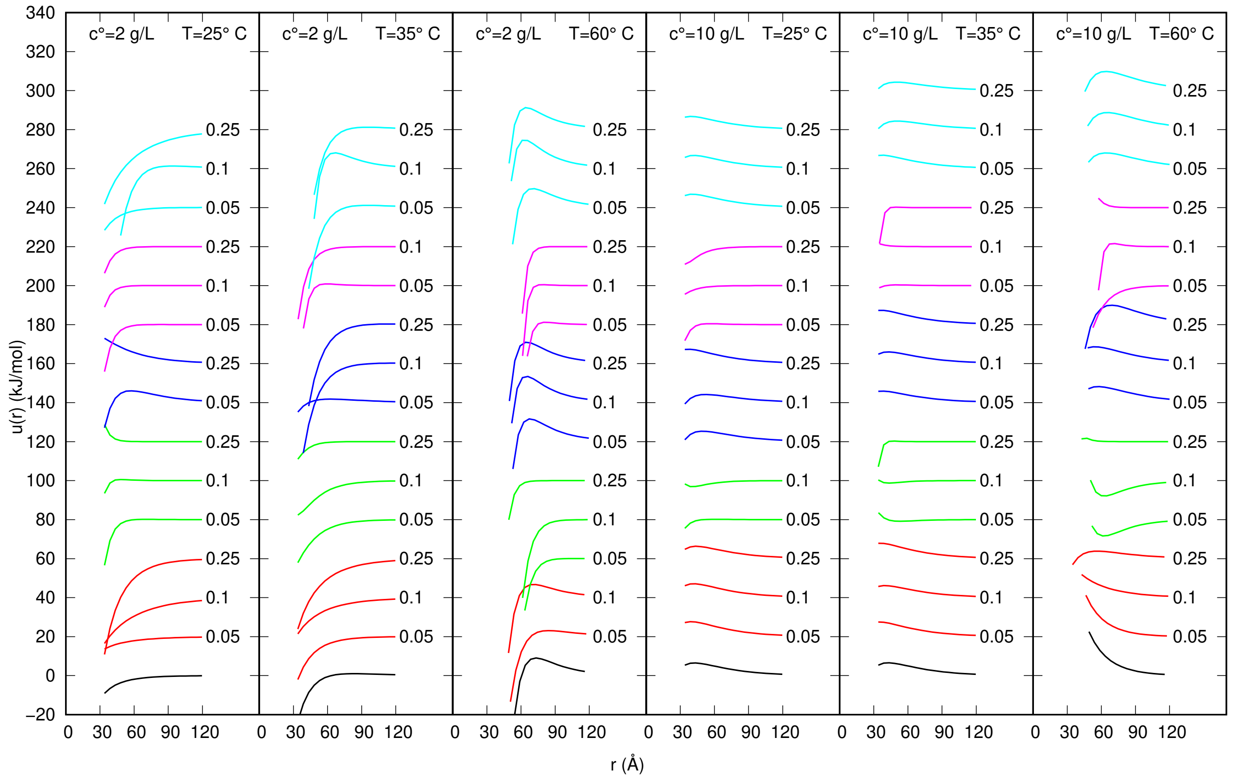

Figure S5: Protein-protein pair potentials obtained by the analysis of SAXS data of MB samples shown in Figure 3. Curve are scaled by a factor 20 kJ/mol for clarity.

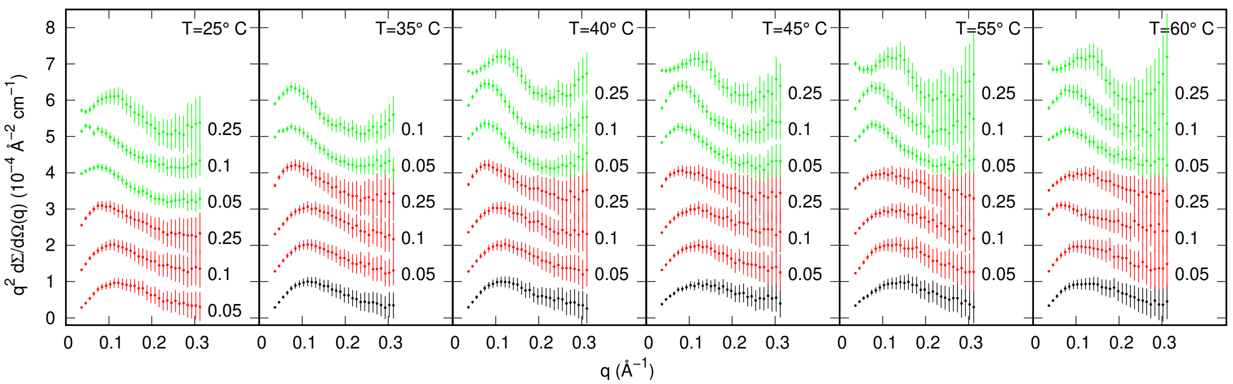

Figure S6: Kratky plots of the experimental SAXS curves of 2 g/L IN in 10 mM phosphate buffer (pH = 3) with and without ExtremoChem modified-sugar. Colors refer to the following conditions: no-modified-sugar (black), EC312 (red), EC101 (green). Whenever present, the modified-sugar concentration is reported on the right side of each curve in molar unit. Each column refers to a fixed temperature, as indicated on the top. Curves in the same column have been scaled by the factor 1 (in the unity of the y-axis). Experimental standard deviations are reported as error bars every 10 points, for clarity.

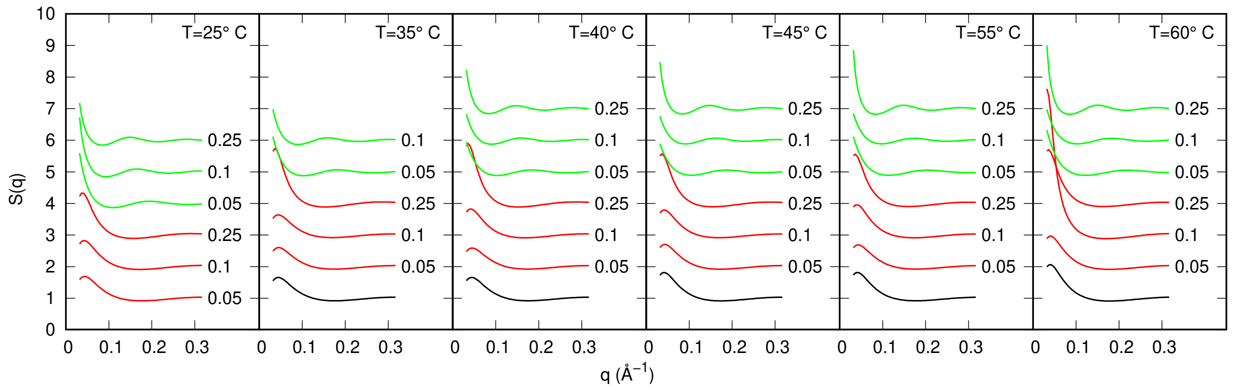

Figure S7: Protein-protein structure factors obtained by the analysis of SAXS data of IN samples shown in Figure 5. Curves are scaled by a factor 1 for clarity.

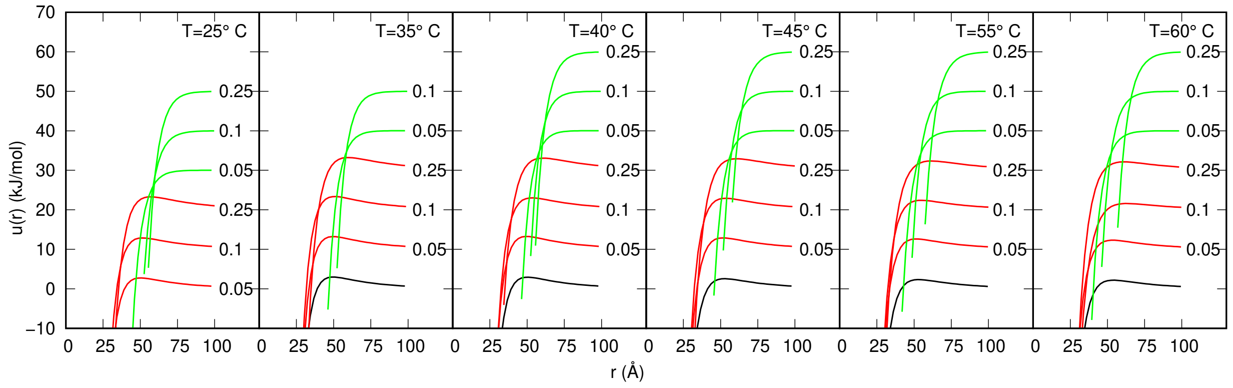

Figure S8: Protein-protein pair potentials obtained by the analysis of SAXS data of IN samples shown in Figure 5. Curve are scaled by a factor 10 kJ/mol for clarity.

References

[1] Ortore, M. G.; Spinozzi, F.; Mariani, P.; Paciaroni, A.; Barbosa, L. R. S.; Amenitsch, H.; Steinhart, M.; Ollivier, J.; Russo, D. Combining structure and dynamics: non-denaturing high-pressure effect on lysozyme in solution. *J. R. Soc. Interface* **2009**, *6*, S619–S634.

[2] Maurus, R.; Overall, C. M.; Bogumil, R.; Luo, Y.; Mauk, A.; Smith, M.; Brayer, G. D. A myoglobin variant with a polar substitution in a conserved hydrophobic cluster in the heme binding pocket. *BBA - Protein Structure and Molecular Enzymology* **1997**, *1341*, 1–13.

[3] Nagao, S.; Osuka, H.; Yamada, T.; Uni, T.; Shomura, Y.; Imai, K.; Higuchi, Y.; Hirota, S. Structural and oxygen binding properties of dimeric horse myoglobin. *Dalton Trans.* **2012**, *41*, 11378–11385.

[4] Moretti, P. Innovative methods to investigate intrinsically disordered proteins by X-ray and neutron scattering techniques. Ph.D. thesis, Life and Environmental Sciences, Ancona, 2019.

[5] O'Donoghue, S. I.; Chang, X.; Abseher, R.; Nilges, M.; Led, J. J. Unraveling the symmetry ambiguity in a hexamer: Calculation of the R6 human insulin structure. *Journal of Biomolecular NMR* **2000**, *16*, 93–108.

[6] Kell, G. S. Density, thermal expansivity, and compressibility of liquid water from 0.deg. to 150.deg.. Correlations and tables for atmospheric pressure and saturation reviewed and expressed on 1968 temperature scale. *J. Chem. Eng. Data* **1975**, *20*, 97–105.

[7] Frauenfelder, H.; Hartmann, H.; Karplus, M.; Kuntz, I. D., Jr; Kuriyan, J.; Parak, F.; Petsko, G. A.; Ringe, D.; Tilton, R. F., Jr; Connolly, M. L. Thermal expansion of a protein. *Biochemistry* **1987**, *26*, 254–261.

[8] Schellart, W. Rheology and density of glucose syrup and honey: Determining their suitability for usage in analogue and fluid dynamic models of geological processes. *Journal of Structural Geology* **2011**, *33*, 1079–1088.

[9] Svergun, D.; Richard, S.; Koch, M. H. J.; Sayers, Z.; Kuprin, S.; Zaccai, G. Protein hydration in solution: experimental observation by X-ray, neutron scattering. *Proc. Natl. Acad. Sci. USA* **1998**, *95*, 2267–2272.

[10] Spinozzi, F.; Mariani, P.; Ortore, M. G. Proteins in binary solvents. *Biophys. Rev.* **2016**, *8*, 87–106.

[11] Jeffrey, P. D.; Coates, J. H. An Equilibrium Ultracentrifuge Study of the Self-Association of Bovine Insulin*. *Biochemistry* **1966**, *5*, 489–498.

[12] Murty, K. G. *Linear programming*; Wiley Subscription Services, Inc., A Wiley Company, 1983.

[13] Spinozzi, F.; Ferrero, C.; Ortore, M. G.; Antolinos, A. D. M.; Mariani, P. GENFIT: software for the analysis of small-angle X-ray and neutron scattering data of macromolecules in-solution. *J. App. Cryst.* **2014**, *47*, 1132–1139.
